# Synthesis, Antimicrobial Evaluation, and Molecular Docking Analysis of Novel Schiff Bases Derived from Isatoic Anhydride and Salicylaldehyde

**DOI:** 10.3390/ijms27020742

**Published:** 2026-01-11

**Authors:** Turgay Tunç, Yaşar Köse

**Affiliations:** Department of Chemical Engineering, Faculty of Engineering and Architecture, Kırşehir Ahi Evran University, 40100 Kırşehir, Türkiye; yasar_kose40@hotmail.com

**Keywords:** Schiff base, salicylaldehyde, spectrophotometric methods, antimicrobial activity, molecular docking

## Abstract

Schiff bases are bioactive compounds that have been synthesized by many researchers in recent years. They may also exhibit strong antimicrobial activities against various pathogenic microorganisms in both medicine and veterinary applications. The synthesis of new Schiff base-derived compounds remains of interest due to the increasing problem of antibiotic-resistance in clinical practice. Seven new Schiff base derivatives were synthesized, and their chemical structures were characterized using FT-IR, ^1^H/^13^C NMR, and LCMS-MS analyses. The antimicrobial activities of thesyntesized compounds against various pathogenic bacteria, yeasts, and fungi were evaluated using the disk-diffusion method, and their MIC values were also determined. In addition, one representative microorganisms from each class were selected for molecular docking studies. IFD analyses were performed for the **4f** and **4g** ligands using the dihydrofolate reductase enzyme. Spectroscopic analyses confirmed the structures of the synthesized compounds, revealing the presence of characteristic imine functionalities and validating the integrity of the molecular frameworks. Antimicrobial assays demonstrated that several derivatives exhibited measurable activity, with compounds **4f** and **4g** showing the most potent effects, displaying MIC values of 32 µg/mL against *B. cereus* and *E. faecalis*, respectively. Molecular docking studies further indicated that both **4f** and **4g** bind efficiently to the DHFR active site. These findings indicate that among the synthesized Schiff base derivatives, compounds **4f** and **4g** exhibit particularly promising antimicrobial activity, warranting further pharmacological evaluation and medicinal chemistry optimization.

## 1. Introduction

Antimicrobial resistance (AMR) has emerged as a significant threat to public health and the global economy since the early 21st century. AMR occurs when microorganisms—including bacteria, fungi, parasites, and viruses—develop resistance to agents used to treat infections, such as antibiotics, through various evolutionary mechanisms [1]. According to recent estimates, approximately 3.57 million of the 4.95 million deaths worldwide in 2019 were attributable to antimicrobial resistance infection. This number is higher than that of causes of death, such as malaria or AIDS [2]. In 2017, the World Health Organization (WHO) identified antibiotic-resistant pathogens from 12 bacterial families as a serious threat to global public health, classifying them into different priority levels (moderate, high, and critical) [3]. Therefore, the search for and development of new antimicrobial agents or alternative therapeutic strategies with more effective mechanisms against antibiotic-resistant microorganisms has become an urgent medical need and a major goal of future research, owing to the lack of effective treatments for pathogens in both human and veterinary medicine [4,5,6].

Salicylaldehyde (2-hydroxybenzaldehyde) is an important organic intermediate with significant potential for the targeted synthesis of various chemicals used in numerous applications, particularly in medicine. This compound contains two distinct reactive functional groups: an aldehyde group and a hydroxyl group. Owing to its bioactive potential, commercial availability, and accessibility, many of its derivatives are widely employed in diverse industrial processes, especially in large-scale pharmaceutical production [7]. Numerous studies have demonstrated the exceptional efficacy of salicylaldehyde as a safe monoaldehyde crosslinker with antifungal, antioxidant, antimycotoxigenic, and chemosensitizing activities [8,9]. In addition, its ability to interact with amine groups confers notable antimicrobial properties. Consequently, the synthesis and investigation of salicylaldehyde derivatives with respect to their diverse biological activities have attracted considerable scientific interest and have found broad applications across multiple research fields [10].

Sirtinol and its derivatives have been widely investigated as inhibitors of sirtuin enzymes (SIRT1 and SIRT2), which are NAD^+^-dependent deacetylases involved in cellular senescence, DNA repair, metabolism, and epigenetic regulation [11,12]. Although these compounds have primarily been studied in eukaryotic systems, particularly in cancer and neurodegenerative disease models, their structural features provide valuable insights for the design of novel bioactive molecules. Therefore, it is of considerable interest to investigate the antimicrobial activity of sitrinol-like compounds by introducing various amine substituents into the synthesized Schiff bases.

Compounds containing a characteristic C=N double bond, formed via the condensation of carbonyl compounds with primary amines under appropriate reaction conditions, are referred to as Schiff bases. They were first synthesized by the German chemist Hugo Schiff in 1860 [13]. Schiff bases possess the general structure R_2_C=NR′. Aryl-substituted Schiff bases are generally more stable and are synthesized more readily than their alkyl-substituted counterparts, owing to the electron-donating effect arising from conjugation of the imine bond with the aromatic ring. In contrast, alkyl-substituted Schiff bases are relatively less stable and typically require longer reaction times for formation. Condensation reactions proceed more rapidly with aldehydes because the carbonyl carbon in aldehydes is sterically less hindered than that in ketones. Furthermore, in ketones, the –R group bonded to the carbonyl carbon donates electron density, thereby reducing the electrophilic character of the carbonyl carbon and, consequently, slowing the reaction rate [14]. Schiff bases and their metal complexes have been extensively reported as promising antimicrobial agents [15,16,17,18,19,20,21]. Schiff bases exhibit diverse biological activities against a broad spectrum of pathogenic microorganisms as well as certain types of tumors. These activities are largely attributed to the presence of the imine (C=N) functional group [22,23,24]. Also, compounds including Schiff bases are used in different chemical fields, especially in catalysts, dyes, pigments, and pharmaceutical industries [25].

**Figure 1:**
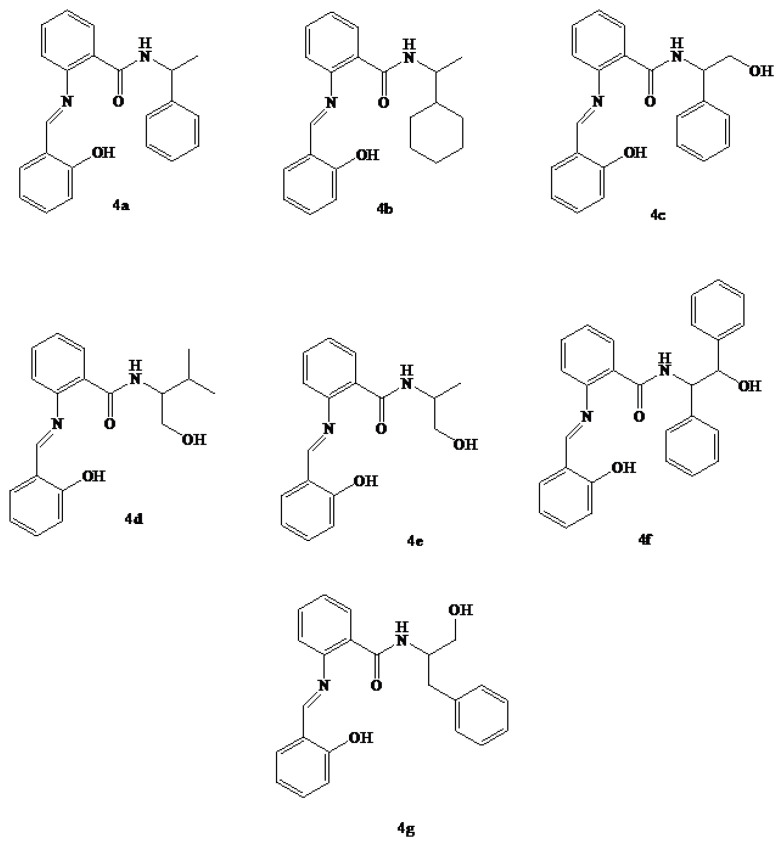
The structure of the synthesized Schiff bases.

In recent years, benzaldehyde-derived compounds have gained considerable interest because of their diverse biological activities and broad applications in medicinal chemistry. Previous studies have demonstrated that Schiff bases derived from benzaldehyde exhibit pronounced antimicrobial properties [26,27,28]. This study reports the synthesis of new salicylaldehyde-based Schiff bases (Figure 1) and the evaluation of their antimicrobial properties. Furthermore, molecular docking studies were employed to investigate the binding modes of the most effective compounds, as shown in **4f** and **4g**.

## 2. Results

### 2.1. Synthesis of Compounds

Schiff bases were synthesized using isatoic anhydride, salicylaldehyde, and seven different amine derivatives. The isolated yields of the synthesized compounds ranged from 30% to 80%. Of these compounds, four contained aromatic substituents, whereas three possessed aliphatic substituents.

The structures of all synthesized compounds were characterized and confirmed by FT-IR, ^1^H and ^13^C NMR, and LCMS-MS spectral analyses. The IR spectra of the Schiff bases exhibited characteristic absorption bands around 3300 cm^−1^ assignable to the NH stretching vibration, and strong bands in the range of 1640–1619 cm^−1^ corresponding to the imine group. The ^1^H-NMR spectra of Schiff bases clearly showed the presence of singlet NH signals at approximately δ 12 ppm (CONH-R). In addition, the formation of the Schiff bases was confirmed by the appearance of a singlet signal attributed to the imine proton (–CH=N–) in the range of δ 7.90–7.60 ppm. Finally, the successful formation of all compounds was further verified by mass spectroscopic data. All relevant spectral figures related to the synthesis results are provided in Supplementary Figures S1–S28.

### 2.2. Antimicrobial Activity

All synthesized Schiff base-derived compounds were first screened for antimicrobial activity against several pathogenic bacteria and yeasts using the disk diffusion method [29]. Subsequently, the minimum inhibitory concentration (MIC) values of the two most active compounds, **4f** and **4g**, were determined (Table 1 and Table 2). Almost all compounds exhibited antimicrobial activity at varying levels; however, only two compounds (**4f** and **4g**) demonstrated promising activity against bacteria, yeasts, and fungi. Compound **4f** showed the lowest MIC value of 32 µg/mL against *Bacillus cereus* 709 ROMA, whereas compound **4g** exhibited an MIC value of 32 µg/mL against *Enterococcus faecalis* ATCC 29212. Interestingly, compound **4f** also displayed relatively low MIC activity (64 µg/mL) against the honeybee pathogen *Ascosphaera apis*. The remaining inhibition zone diameters and MIC values are presented in Table 1 and Table 2. Overall, compounds containing aromatic substituents exhibited stronger antimicrobial activity than those containing aliphatic groups.

### 2.3. Molecular Docking

Docking analyses were performed using one representative organism from each group: a Gram-negative bacterium (*E. coli*), a Gram-positive bacterium (*S. aureus*), and the yeast *C. albicans*. Dihydrofolate reductase (DHFR) enzyme, conserved in all three species, was selected as the target. The crystal structures or the IFD analysis were as follows: *E. coli* dihydrofolate reductase (DHFR) in complex with trimethoprim (TOP) **(PDB ID: 7NAE)**, *S. aureus* (DHFR) in complex with trimethoprim (PDB ID: 2W9S), and *C. albicans* DHFR complexed with 5-chloryl-2,4,6-quinazolinetriamıne (CLZ) (PDB ID: 1M78).

**Figure 2:**
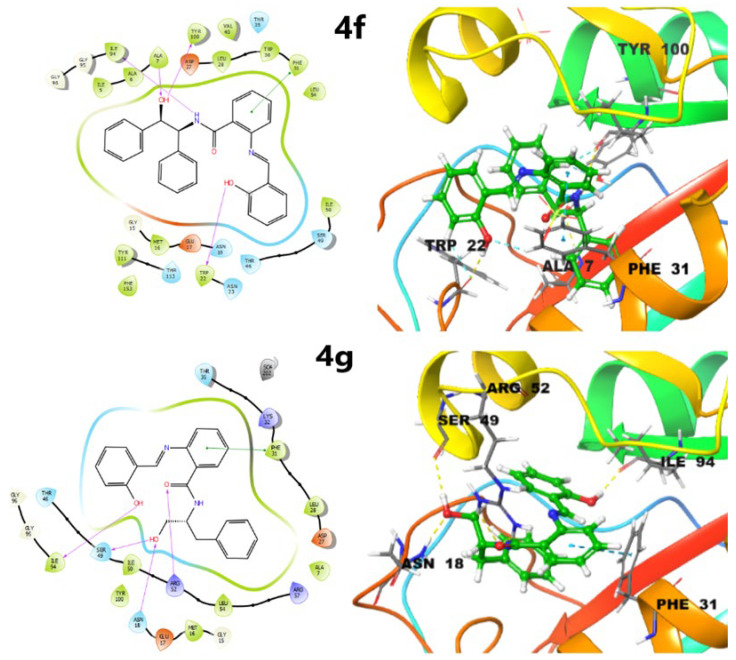
Binding interactions of **4f** and **4g**: 2D interactions (**left**) and 3D interactions (**right**) in the active site of *E. coli* DHFR enzyme (PDB ID: 7NAE).

**Figure 3:**
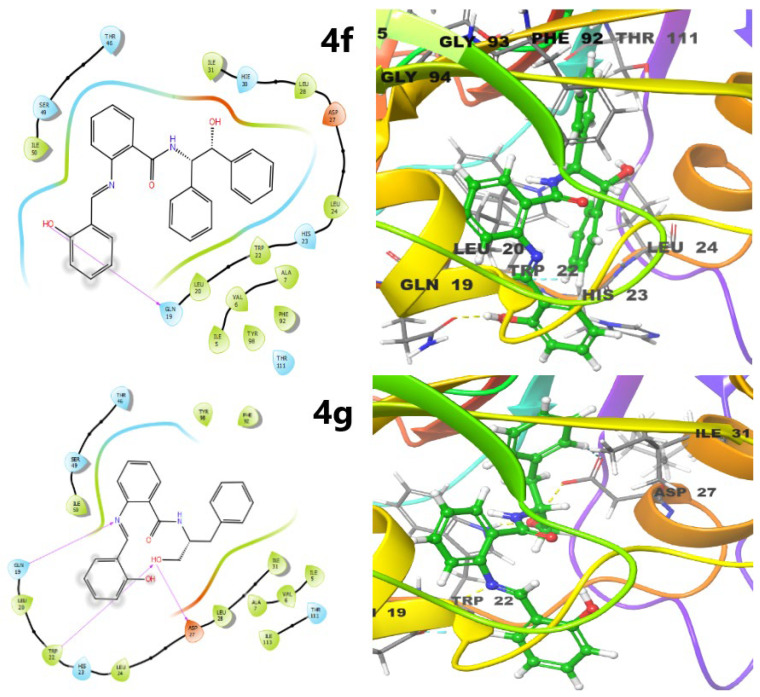
Binding interactions of **4f** and **4g**: 2D interactions (**left**) and 3D interactions (**right**) in the active site of *S. aureus* DHFR enzyme (PDB ID: 2W95).

**Figure 4:**
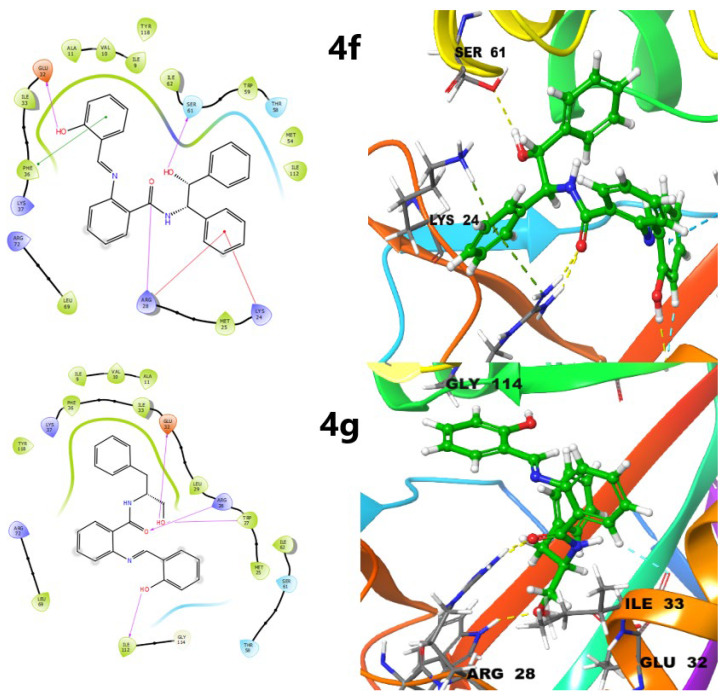
Binding interactions of **4f** and **4g**: 2D interactions (**left**) and 3D interactions (**right**) in the active site of *C. albicans* DHFR enzyme (PDB ID: 1M78).

The IFD docking analyses were carried out for compounds **4f** and **4g**, which exhibited the highest antimicrobial activity. The best IFD docking score and ΔG MMBSA binding energy values were listed in Table 3, while the corresponding intermolecular interactions were presented in Table 4. The best docking poses of **4f** and **4g** were illustrated in Figure 2, Figure 3 and Figure 4. Both compounds displayed comparable docking scores and similar interaction profiles within the active sites, indicating efficient binding to DHFR that is comparable to that of co-crystallized ligands.

### 2.4. ADME Analysis

ADME (acronym for absorption, distribution, metabolism, and excretion) describes the disposition of a pharmaceutical compound within an organism. Pharmacokinetic parameters calculated by the Qikpro module of **4f** and **4g** compounds are shown in Figure 4 or Table 5. Further, **4f** and **4g** showed compliance with the Lipinski and the Jorgensen rule, with the only exception of **4f** logPo/w: 5.95. These indicate that **4f** and **4g** compounds might have drug-like properties. Madin–Darby canine kidney (MDCK) cell permeability in nm/sec is a reliable model for the calculation of oral drug absorption for the gut–blood barrier [30]. PMDCK values of **4f** and **4g** were between which within limits. Based on the predicted values for brain/blood partition coefficient (logBB) and Central Nervous System (CNS) activity, **4f** and **4g** might be able to penetrate the CNS. IC50 value for blockage of HERG K + channels (logHERG) was very low, which was below the limits. Skin permeability (logKp) and the binding to human serum albumin (PlogKhsa) predicted values showed promising results. The magnitude of absorption was expressed by the percentage of absorption. The human oral absorption values of two compounds were good based on ADME analysis.

## 3. Discussion

Spectroscopic confirmation of imine (–CH=N–) formation—evidenced by IR absorption bands in the range of ~1620–1640 cm^−1^ and characteristic imine proton signals in the ^1^H NMR spectra—indicates that the azomethine moiety is preserved in the final molecules and remains available for intermolecular interactions. The imine nitrogen and the adjacent conjugated framework act as electron-donating and electron-accepting motifs, facilitating hydrogen bonding, coordination interactions with metal centers, and π–π stacking interactions with aromatic residues of protein targets [31]. Consequently, these structural features, which are present in the synthesized compounds, are frequently invoked to explain the biochemical activity of Schiff bases in enzymatic systems. The functional role of the azomethine group was particularly evident in compounds **4f** and **4g**, as supported by both spectroscopic characterization and experimental biological studies.

The present study demonstrates that isatoic anhydride-derived Schiff bases exhibit measurable antimicrobial activity against Gram-positive and Gram-negative bacteria, yeasts, and a filamentous fungal strain (*A. apis*). Among the tested compounds, **4f** and **4g** showed relatively strong activity against several of the evaluated pathogens. Consistently, the molecular docking results supported the experimental findings, in agreement with recent reports on the bioactivity of Schiff bases. Moreover, previous studies have shown that conjugated aromatic systems, which are present in synthesized compounds, enhance membrane affinity and/or facilitate target interactions in microbial cells [32].

The MM-GBSA ΔG binding energy obtained from molecular docking analysis predicts the strength of interaction between a compound and its target protein. Theoretical results from induced-fit docking (IFD) analysis indicate a correlation between in vitro antimicrobial activity and MM-GBSA binding free energy values. In the present study, compounds **4f** and **4g**, which exhibited higher antimicrobial activity, also showed stronger binding affinities, as reflected by more negative MM-GBSA ΔG values. The calculations suggest that these enhanced interactions are mainly driven by non-covalent forces, particularly hydrogen bonding and π–π stacking interactions. MM-GBSA values also support structure–activity relationships (SAR), helping explain why compounds like **4f** and **4g** are more active. SAR analysis derived from the dataset offers two practical insights for lead optimization. Firstly, substituents that enhance planarity and conjugation (such as aromatic or heteroaromatic rings) tend to improve biological activity owing to increased binding affinity and membrane permeability, whereas highly flexible aliphatic chains correlate with weaker potency. Secondly, the presence of hydrogen-bond donors or acceptors in positions capable of forming directed interactions with DHFR active-site residues appears beneficial in terms of biological activity. These SAR motifs are consistent with the broader literature on Schiff base scaffolds and can guide rational modifications, such as heteroaryl replacement, strategic introduction of electron-withdrawing or electron-donating groups, or rigidifying linkers, to enhance potency and selectivity [11].

The calculated ADME parameters indicate that compounds **4f** and **4g** generally comply with the drug-likeness criteria proposed by Lipinski and Jorgensen, with the notable exception of the elevated logP value observed for compound **4f**. Although increased lipophilicity may enhance membrane permeability and biological activity, it can also reduce aqueous solubility and increase non-specific binding and metabolic clearance [33]. Therefore, optimization strategies aimed at balancing lipophilicity—such as the introduction of polar substituents or the use of prodrug approaches—should be considered during lead optimization. Moreover, the predicted low hERG liability and acceptable MDCK permeability are encouraging; however, these silico findings require experimental validation, including microsomal stability assays, plasma protein binding studies, hERG patch-clamp experiments, and in vivo pharmacokinetic evaluations. Overall, while in silico ADME prediction represents a valuable early-stage screening tool, it cannot replace comprehensive empirical ADME and toxicological assessments.

## 4. Materials and Methods

### 4.1. Materials

All solvents and reagents used in this study were purchased commercially.

### 4.2. General Synthesis Method of the New Schiff Bases

**Scheme 1:**
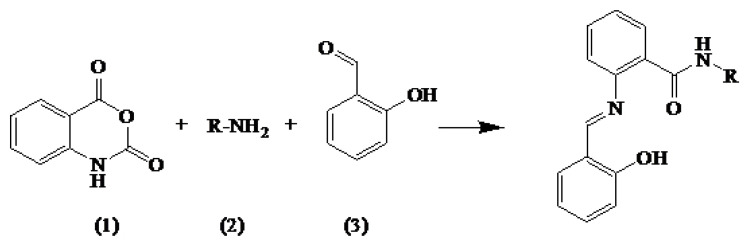
Synthesis of Schiff bases with isatoic anhydride (1), amines (2), and aldehyde (3).

The synthesis pathway of the Schiff bases derived from isatoic anhydride is illustrated in Scheme 1. Isatoic anhydride (15 mmol) and phenylamine (20 mmol) were dissolved in toluene/ethyl acetate (each 20 mL). The mixture was stirred at 40 °C for 60 min. After that, the reaction mixture was filtered and recrystallized from ethanol to obtain the pure compound for each. The intermediate compound (3 mmol) was dissolved in ethanol, and then salicylaldehyde (3 mmol) was added and refluxed for 2 h. The mixture was then cooled at room temperature, and the yellow product was precipitated in a methanol–water mixture (4:1), filtered, and crystallized from ethanol.

### 4.3. Spectral Data of Synthesized Compounds

The spectral data for each compound was provided below by indicating their names from **4a** to **4g**.

For **4a**, the compound was obtained as a yellow powder in 40% yield (0.41 g); m.p.: 135–137 °C. IR (cm^−1^): 3312 (–NH stretch), 3061 (aromatic –CH), 2969 (aliphatic –CH), 1639 (imine –HC=N), 1593 (–C=C–), 1282. ^1^H NMR (400 MHz, dmso-d_6_) δ 12.34 (s, 1H), 8.92 (d, *J* = 8.44 Hz, 1H), 8.85 (s, 1H), 7.64 (d, *J* = 8.0 Hz, 1H), 7.53 (t, *J* = 5.9 Hz, 2H), 7.37 (m, *J* = 6.42 Hz, 5H), 7.21 (m, *J* = 10.37 Hz, 3H), 6.95 (d, *J* = 3.64 Hz, 2H), 5.11 (m, *J* = 14.36 Hz, 1H), 1.43 (m, *J* = 7.2, 7.2, 5.84, 3H). ^13^C NMR (100 MHz, dmso-d_6_) δ 166.90, 163.96, 160.70, 147.15, 145.18, 134.22, 132.34, 131.54, 128.89, 127.05, 126.72, 126.67, 120.41, 119.81, 119.73, 117.40, 49.09, 23.04. LC-MS (*m*/*z*): C_22_H_20_N_2_O_2_ (calc. 344.41), 345.10 [M + H].

For **4b**, the compound was obtained as a yellow powder in 35% yield (0.37 g); m.p.: 170–172 °C. IR (cm^−1^): 3272 (–NH stretch), 2970 (aromatic –CH), 2928 (aliphatic –CH), 1632 (imine –HC=N), 1528 (–C=C–), 1282. ^1^H NMR (400 MHz, dmso-d_6_) δ 12.25 (s, 1H), 10.25 (s, 1H), 8.81 (s, 1H), 8.20 (d, *J* = 8.76 Hz, 1H), 7.66 (d, *J* = 8.48 Hz, 1H), 7.50 (m, *J* = 9.82 Hz, 2H), 7.32 (d, *J* = 7.24 Hz, 2H), 6.95 (s, 1H), 6.63 (m, *J* = 16 Hz, 1H), 2.48 (s, 3H), 1.90 (s, 1H), 1.61 (m, *J* = 11.07 Hz, 5H), 1.02 (m, *J* =4.57 Hz, 6H). ^13^C NMR (100 MHz, dmso-d_6_) δ 166.91, 163.48, 160.67, 147.16, 134.15, 132.70, 132.00, 131.26, 128.99, 126.94, 120.40, 119.72, 117.35, 49.82, 42.96, 29.73, 26.38, 18.19. LC-MS (*m*/*z*): C_22_H_26_N_2_O_2_ (calc. 350.45), 351.95 [M + H].

For **4c**, the compound was obtained as a yellow powder in 45% yield (0.49 g); m.p.: 168–170 °C. IR (cm^−1^): 3287 (–NH stretch), 3061 (aromatic –CH), 2931 (aliphatic –CH), 1619 (imine –HC=N), 1538 (–C=C–), 1280. ^1^H NMR (400 MHz, dmso-d_6_) δ 12.01 (s, 1H), 9.03 (s, 1H), 8.87 (s, 1H), 7.73 (m, *J* = 10.70 Hz, 3H), 7.37 (m, *J* = 5.72 Hz, 7H), 6.97 (m, *J* = 7.62 Hz, 3H), 6.68 (s, 1H), 4.99 (m, *J* = 28.76 Hz, 2H), 2.50 (s, 1H). ^13^C NMR (100 MHz, dmso-d_6_) δ 167.03, 163.24, 160.51, 147.90, 141.78, 137.11, 134.27, 132.38, 131.90, 131.20, 128.67, 127.40, 120.69, 120.05, 119.84, 117.92, 65.25, 56.16. LC-MS (*m*/*z*): C_22_H_20_N_2_O_3_ (calc. 360.41), 361.10 [M + H].

For **4d**, the compound **4d** was obtained as a yellow powder in 42% yield (0.41 g); m.p.: 209–211 °C. IR (cm^−1^): 3435 (–OH stretch), 3273 (–NH stretch), 3063 (aromatic –CH), 2972 (aliphatic –CH), 1620 (imine –HC=N), 1537 (–C=C–), 1284. ^1^H NMR (400 MHz, dmso-d_6_) δ 12.05 (s, 1H), 8.85 (s, 1H), 8.27 (d, *J* = 9.0 Hz, 1H), 7.75 (d, *J* = 6.92 Hz, 1H), 7.68 (d, *J* = 6.96 Hz, 1H), 7.54 (t, *J* = 14.04 Hz, 1H), 7.36 (m, *J* = 10.35 Hz, 3H), 6.97 (d, *J* = 4.1 Hz, 2H), 4.58 (s, 1H), 3.83 (s, 1H), 2.51 (s, 1H), 1.91 (m, *J* = 21.28 Hz, 1H), 0.83 (q, *J* = 5.49, 6H). ^13^C NMR (100 MHz, dmso-d_6_) δ 166.67, 162.38, 159.85, 146.91, 133.52, 131.13, 130.87, 128.75, 126.19, 119.92, 119.04, 116.62, 61.19, 56.06, 28.15, 19.61, 18.10. LC-MS (*m*/*z*): C_19_H_22_N_2_O_3_ (calc. 326.39), 327.10 [M + H].

For **4e**, the compound was obtained as a yellow powder in 30% yield (0.27 g); m.p.: 164–166 °C. IR (cm^−1^): 3440 (–OH stretch), 3279 (–NH stretch), 3054 (aromatic –CH), 2975 (aliphatic –CH), 1619 (imine –HC=N), 1517 (–C=C–), 1282. ^1^H NMR (400 MHz, dmso-d_6_) δ 12.34 (s, 1H), 8.89 (d, *J* = 4.1 Hz, 1H), 8.27 (s, 1H), 7.73 (d, *J* = 7.92 Hz, 1H), 7.59 (d, *J* = 7.64 Hz, 1H), 7.53 (d, *J* = 7.64 Hz, 1H), 7.40 (m, *J* = 10.24 Hz, 3H), 6.98 (q, *J* = 6.1 Hz, 2H), 4.72 (d, *J* = 8.08 Hz, 1H), 3.96 (q, *J* = 11.04 Hz, 1H), 2.50 (s, 2H), 1.09 (t, *J* = 10.80 Hz, 4H). ^13^C NMR (100 MHz, dmso-d_6_) δ 167.16, 163.57, 160.68, 146.95, 134.20, 132.55, 131.47, 129.18, 127.06, 120.40, 119.54, 117.35, 64.99, 47.72, 17.74. LC-MS (*m*/*z*): C_17_H_18_N_2_O_3_ (calc. 298.34), 299.10 [M + H].

For **4f**, the compound was obtained as a yellow powder in 52% yield (0.68 g); m.p.: 174–176 °C. IR (cm^−1^): 3293 (–NH stretch), 3061 (aromatic –CH), 2945 (aliphatic –CH), 1637 (imine –HC=N), 1537 (–C=C–), 1284. ^1^H NMR (400 MHz, dmso-d_6_) δ 11.76 (s, 1H), 9.14 (d, *J* = 9.16 Hz, 1H), 8.77 (s, 1H), 7.68 (d, *J* = 7.76 Hz, 1H), 7.52 (t, *J* = 15.16 Hz, 1H), 7.44 (d, *J* = 3.26 Hz, 2H), 7.31 (t, *J* = 5.09 Hz, 3H), 7.24 (m, *J* = 4.56 Hz, 6H), 7.14 (t, *J* = 4.51 Hz, 4H), 6.98 (d, *J* = 4.01 Hz, 2H), 5.55 (d, *J* = 5.04 Hz, 1H), 5.26 (t, *J* = 15.92 Hz, 1H), 4.89 (t, *J* = 11.80, 1H), 3.38 (s, 2H), 2.50 (s, 1H). ^13^C NMR (100 MHz, dmso-d_6_) δ 165.25, 161.97, 159.74, 147.51, 142.78, 140.14, 133.61, 131.31, 130.90, 129.76, 128.74, 1285.25, 127.53, 127.37, 129.79, 126.60, 126.08, 120.18, 119.15, 116.65, 74.96, 58.59. LC-MS (*m*/*z*): C_28_H_24_N_2_O_3_ (calc. 436.50), 437.10 [M + H].

For **4g**, the compound was obtained as a yellow powder in 82% yield (0.92 g); m.p.: 190–192 °C. IR (cm^−1^): 3447 (–OH stretch), 3282 (–NH stretch), 3061 (aromatic –CH), 2942 (aliphatic –CH), 1620 (imine –HC=N), 1533 (–C=C–), 1280. ^1^H NMR (400 MHz, dmso-d_6_) δ 12.22 (s, 1H), 8.86 (d, *J* = 3.52 Hz, 1H), 8.47 (d, *J* = 4.88 Hz, 1H), 7.71 (d, *J* = 6.40 Hz, 1H), 7.50 (m, *J* = 6.81 Hz, 3H), 7.37 (m, *J* = 11.04 Hz, 2H), 7.21 (d, *J* = 3.40 Hz, 5H), 6.98 (t, *J* = 4.96 Hz, 2H), 4.86 (s, 1H), 4.15 (s, 1H), 2.96 (m, *J* = 22.68 Hz, 1H), 2.74 (m, *J* = 22.20 Hz, 1H), 2.50 (s, 1H). ^13^C NMR (100 MHz, dmso-d_6_) δ 166.43, 162.42, 159.94, 146.54, 139.10, 133.52, 131.54, 130.91, 129.05, 128.44, 128.01, 126.06, 125.95, 119.87, 118.89, 116.67, 62.36, 52.71, 36.41. LC-MS (*m*/*z*): C_23_H_22_N_2_O_3_ (calc. 374.43), 375.05 [M + H].

### 4.4. Antimicrobial Susceptibility Testing

#### 4.4.1. Test Microorganisms

For antimicrobial activity assays, eight bacteria, two yeasts, and one filamentous fungus were selected. The test microorganisms were determined as *Escherichia coli* ATCC 25922, *Yersinia pseudotuberculosis* ATCC 911, *Pseudomonas aeruginosa* ATCC 43288, *Enterococcus faecalis* ATCC 29212, *Listeria monocytogenes* ATCC 43251, *Staphylococcus aureus* ATCC 25923, *Bacillus cereus* 709 Roma, *B. subtilis* subs. *spizizenii* ATCC 6633, *Candida albicans* ATCC 60193, *Saccharomyces cerevisiae* RSKK 251, and *Ascosphaera apis* 24.

#### 4.4.2. Antimicrobial Assays

For antibacterial assays, the standard Kirby–Bauer disk diffusion method was used [29]. All bacterial strains were inoculated in 3 mL of nutrient broth from stock cultures and incubated at 37 °C overnight. After that, bacterial cultures were adjusted to 0.5 McFarland turbidity by a spectrophotometer using sterile saline solution. The adjusted bacterial suspensions were equally applied onto all sides of Mueller–Hinton Agar (MHA) using a sterile swab. Standard disks (6 mm diameter) were loaded with 100 µL of chemical solution (dissolved in methanol) and placed onto the agar and incubated at 37 °C for 24 h. After incubation, the antibacterial activity was recorded by measuring zone diameters. Ampicillin disk (25 µg) was used as a positive control, and methanol was used as a negative control.

For anti-yeast activity assays, *Candida* and *Saccharomyces* strains were grown as described above, but PDB and YPS (Yeast peptone dextrose agar) were used as medium. Incubation conditions were set at 25 °C for 48 h. Fluconazole (25 µg) was used as a positive control, and methanol was used as a negative control [29].

*Ascosphaera apis* (Maassen ex Claussen) LS Olive and Spiltoir (1955) is an important pathogen of honeybee larvae and causes chalk-brood disease in honeybee colonies worldwide [34]. In this study, we also tested all compounds against this pathogen. *A. apis* isolate 24 was grown on MY20 agar (20% dextrose agar) and was incubated at 32 °C for 20 days in the dark [35]. Spores were harvested as described above and used for tests. To test the chemicals against *A. apis*, the agar dilution method was performed [36]. The inhibition of the hypha (zone inhibition) was determined. Briefly, a spore suspension of 9 × 10^7^ spores/mL was spread onto MY20 agar and left to dry. After that, 0.5 mL of each chemical was placed in a central hole (7 mm) by adjusting the appropriate concentration and incubated at 32 °C in the dark for 4–5 days. After that, the fungal growth (inhibition zone) was measured. Fluconazole (128 µg/mL) was used as a positive control, and methanol was used as a negative control. All tests were repeated three times.

#### 4.4.3. Minimum Inhibitory Concentration (MIC)

The most effective chemicals (**4f** and **4g**) were selected based on screening initial tests and were used for MIC calculations. A broth microdilution method was used for MIC calculations according to the Clinical and Laboratory Standards Institute (CLSI) guidelines [29]. For bacterial strains, suspensions were grown until the exponential growth phase, and the bacterial concentrations for each pathogen were adjusted to 1 × 10^6^ cfu/mL. After that, 100 µL of a two-fold diluted chemical dissolved in methanol with Mueller–Hinton Broth was introduced to 96-well microtiter plates, and 100 µL of the bacterial suspensions were added to each well containing the test substances. The blank well was used as sterility control. The concentrations of test substances ranged from 512 to 2 µg/mL. Ampicillin was used as a positive control. After incubation for 24 h at 37 °C, the turbidity was observed as an indication of growth, and the lowest concentration inhibiting the visible growth of bacteria was recorded as the MIC [37]. All the experiments were performed in triplicate.

To calculate MICs of **4f** and **4g** against yeast strains, the same method as described above was used with small modifications. YPS (yeast peptone dextrose broth) was used as a medium. Incubation conditions were set at 25 °C for 48 h. Fluconazole was used as a positive control, and the blank well was used as a sterility control. All tests were performed in triplicate [29].

For MIC calculation of **4f** and **4g** against *A. apis*, the agar dilution method was used [38]. The chemicals **4f** and **4g** (25, 50, 250, and 500 µL dissolved in 500 µL methanol) were slowly mixed with 100 mL of MY20 agar medium and poured into Petri dishes (90 ×15 mm). The final concentrations were adjusted to 250, 500, 2500, and 5000 µL/L. The medium MY20 agar with 5.000 µL methanol was used negative control. For all experiments, the relevant medium without anything was used to test sterility control. Finally, 8 mm diameter agar was removed from actively growing *A. apis* colonies and put onto agar medium containing fungal extracts and incubated at 32 °C for 20 days in the dark. At the end of the incubation, the MIC value was detected based on the growing status.

### 4.5. Molecular Docking Analyses

#### 4.5.1. Calculation Methods

The steps in the calculation methodology for docking analysis are as follows: selection of target enzymes, ligand preparation, protein preparation, induced field docking, molecular dynamics simulation, trajectory analysis, and ADME prediction. Schrödinger software (Release, 2020-3) and various embedded modules have been used for all calculations, such as Desmond v6.3, Epic v5.3, glide v8.8, Jaguar v10.9, Maestro v12.5, QuikProp v6.5, LigPrep, and MM-GBSA [39,40].

#### 4.5.2. Ligand Preparation

Two-dimensional structures of the constituents were retrieved from the PubChem database. The 3D models of the ligands, possible ionization, and tautomeric states of the constituents were prepared by LigPrep v2.3 module using OPLS_2005 force field. Extra optimization of the structures was performed with the Jaguar v 10.9 program using the DFT/B3LYP-3D/6-31G (d,p) method.

#### 4.5.3. Protein Preparation

X-ray crystal structures of the target were imported from the RCSB Protein Data Bank. The enzymes were prepared using the Protein Preparation Wizard module of Maestro 12.5. In this process, the protein structures were corrected by adding hydrogen atoms and missing residues, assigning bond orders and bond length, creating disulfide bonds, fixing the charges, refining the loop with Prime, removing the water molecules, and, finally, minimizing by using the OPLS-2005force field at a pH of 7.4. Ionization and tautomeric states were generated by Epik v5.3, and the proton orientations were set by PROPKA. Restrained minimization was run with convergence of heavy atoms to an RMSD of 0.3 Å.

#### 4.5.4. Molecular Docking

Schrödinger IFD protocol was used for the IFD-docking calculations [38]. The receptor grid center was specified from the bound cognate ligand with a cubic grid, and the side chains were automatically trimmed according to the B factor. Default parameters were used for receptor van der Waals scaling factor 0.70 and ligand van der Waals scaling factor 0. All residues within 5.0 Å of ligand poses were refined using the Prime molecular dynamics module to allow for binding domain flexibility. Glide SP protocol with OPLS_2005 force field was used for the redocking step into the top 20 receptor structures generated within 30 kcal/mol of the best structure by the Prime refinements. The docking method was verified using the redocking test. Cognate ligands were redocked into corresponding binding pockets. RMSD values of the redocked cognate ligands were observed to be in the range of 0.26–0.46 Å, confirming the accuracy and feasibility of the docking method.

#### 4.5.5. Binding Free Energy Calculations

The relative binding free energy of the protein–ligand complexes was calculated by the molecular mechanics-generalized born surface area (MM-GBSA) method using the Prime program of Schrodinger software 2025-1. The OPLS_2005 force field in the VSGB solvent model was used to calculate energies. The free energy of the complexes was calculated using the equation below.

MMGBSA ΔG Bind = EComplex − EReceptor − Eligand

While MMGBSA represents molecular mechanics energies combined with the generalized born and surface area continuum solvation, ΔG bind represents the calculated relative free energy of both the ligand and receptor strain energy, EComplex represents the MM/GBSA energy of the minimized complex, EReceptor represents the mean MM/GBSA energy of protein (unbound, minimized) without ligand, and Eligand represents the MM/GBSA energy of the ligand after removing it from the complex [41].

#### 4.5.6. ADME Analysis

Qikprop was used to calculate pharmacokinetic properties of the compounds (QikProp, version 6.5, Schrödinger, LLC, New York, NY, USA, 2020-3). Rule of Five (Ro5), known as Lipinski’s Rule (MW < 500, logPo/w < 5, donorHB ≤ 5, accptHB ≤ 10), and Rule of Three (Ro3), known as Jorgensen’s Rule (logS > −5.7, PCaco > 22 nm/s, total primary metabolites < 7), were determined. The other calculated pharmacokinetic properties are LogHERG, IC_50_ value for blockage, (<25 poor, >500 great); PCaco, predicted apparent Caco-2 cell permeability in m/sec (<25 poor, >500 great); LogBB, predicted brain/blood partition coefficient, (−3.0/1.2); **P_MDCK_**, predicted apparent MDCK cell permeability in nm/sec (<25 poor >500 great); LogKp, skin permeability (−8.0/−1.0); PLogKhsa, binding to human serum albumin (−1.5/1.5); CNS, Central Nervous System activity (−2/2); and % ABS, Percent Human Oral Absorption (>80%/<25%).

## 5. Conclusions

Antimicrobial resistance, encompassing antibacterial and antifungal resistance, represents a major global challenge in the treatment of infectious diseases, with drug-resistant bacterial and fungal infections accounting for substantial morbidity and mortality worldwide. The evolutionary emergence of resistance in microorganisms appears inevitable; therefore, the development of more effective, affordable, and readily accessible antimicrobial agents remains a critical research priority. The Schiff base-containing compounds synthesized in this study, particularly compounds **4f** and **4g**, demonstrated promising antimicrobial activity against a range of bacterial and fungal pathogens.

Induced-fit docking (IFD) analysis revealed that compounds **4f** and **4g** exhibit strong inhibitory potential against dihydrofolate reductase (DHFR). Furthermore, MM-GBSA binding free energy calculations indicate that ligands **4f** and **4g** bind more favorably to the DHFR active site than the reference inhibitors trimethoprim (TMP) and 5-chloro-2,4,6-quinazolinetriamine (CLZ). The most important non-covalent interactions between the compound and DHFR enzyme are hydrogen bonding and pi-pi-stacking. These findings suggest a higher binding affinity of the synthesized compounds toward DHFR; however, molecular dynamics simulations are warranted to further assess the stability and persistence of these ligand–enzyme interactions within the active site.

In addition, to further enhance the antimicrobial efficacy of these compounds, rational structural modifications should be considered. These include the introduction of electron-withdrawing or electron-donating substituents on the aromatic rings to modulate electronic distribution, optimization of the steric environment around the azomethine (C=N) linkage to improve active-site accommodation, and incorporation of additional hydrogen bond donors or acceptors to strengthen key interactions with DHFR.

## Figures and Tables

**Table 1:** Antimicrobial susceptibility assay results.

No	Diameter of Inhibition Zone (mm)										
	EC	YP	PA	EF	LM	SA	BC	BS	CA	SC	AA
**4f**	25.2 ± 2.8	0 ± 0	19.6 ± 3.4	15.5 ± 1.5	18 ± 2	26.7 ± 3.7	17.3 ± 1.3	30.8 ± 3,4	26.2 ± 3.2	0 ± 0	23.1 ± 4.7
**4g**	20.9 ± 1.9	10.4 ± 2.2	16.4 ± 2.6	26.8 ± 4.8	14.1 ± 1.3	10.1 ± 1.1	22.5 ± 1.5	21.6 ± 0.8	12.3 ± 0.9	0 ± 0	9.6 ± 0.2
**4c**	18.3 ± 1.9	9.5 ± 2.5	13.3 ± 2.9	21.6 ± 2.6	18.4 ± 0.8	10.3 ± 2.3	9.7 ± 3.3	19.3 ± 1.9	14.2 ± 1.2	2.6 ± 1.2	10.7 ± 1.3
**4a**	5.7 ± 2.5	10.5 ± 2.5	8.5 ± 1.5	14.2 ± 1.4	8.8 ± 0.6	0 ± 0	9.7 ± 0.7	9.1 ± 0.9	0 ± 0	0 ± 0	4.6 ± 1.2
**4b**	8.3 ± 1.5	0.5 ± 0	3 ± 0	1.8 ± 0.8	1 ± 0	3.6 ± 1.6	7.5 ± 2.5	7.1 ± 1.1	2.8 ± 1.2	0 ± 0	1.6 ± 0.6
**4d**	1.2 ± 1.2	0 ± 0	7.6 ± 1.7	4.6 ± 0.6	9.5 ± 2.5	0 ± 0	10.7 ± 1.7	17.5 ± 2.5	12.5 ± 1.5	1.1 ± 0.3	6.8 ± 2.2
**4e**	0.9 ± 0.3	0 ± 0	13.1 ± 1.6	8.2 ± 0.1	12.4 ± 2.6	2.3 ± 0.9	11.4 ± 4.6	11.6 ± 2.8	10.2 ± 1.8	1 ± 0	5.6 ± 2.4
PC	25.4 ± 4.2	-	30.45 ± 1.5	22.2 ± 1.8	27.7 ± 5.3	25.9 ± 8	42.66 ± 7.9	40.9 ± 6.7	24.33 ± 6.4	-	21 ± 9.6

Diameter of inhibition zone (mm) was presented as means (±SD), including a 6 mm paper disk after three replications. EC: *Escherichia coli* ATCC 25922; YP: *Yersinia pseudotuberculosis* ATCC 911; PA: *Pseudomonas aeruginosa* ATCC 43288; EF: *Enterococcus faecalis* ATCC 29212; LM: *Listeria monocytogenes* ATCC 43251; SA: *Staphylococcus aureus* ATCC 25923; BC: *Bacillus cereus* 709 Roma; BS: *B. subtilis* subs. *spizizenii* ATCC 6633; CA: *Candida albicans* ATCC 60193; SC: *Saccharomyces cerevisiae* RSKK 251; AA: *Ascosphaera apis* 24. PC: positive control. Ampicillin and Fluconazole were used as positive controls for bacteria and fungi, respectively. The hundred digits for all measurements (mm) were rounded.

**Table 2:** MIC (µg/mL) values of the selected compounds.

Compound	MIC (µg/mL)										
	EC	YP	PA	EF	LM	SA	BC	BS	CA	SC	AA
**4f**	64	NA	128	128	128	64	64	32	64	NA	64
**4g**	64	128	64	32	256	256	128	64	256	NA	256
PC	32	NA	32	64	32	32	32	16	32	64	50

EC: *Escherichia coli* ATCC 25922; YP: *Yersinia pseudotuberculosis* ATCC 911; PA: *Pseudomonas aeruginosa* ATCC 43288; EF: *Enterococcus faecalis* ATCC 29212; LM: *Listeria monocytogenes* ATCC 43251; SA: *Staphylococcus aureus* ATCC 25923; BC: *Bacillus cereus* 709 Roma; BS: *B. subtilis* subs. *spizizenii* ATCC 6633; CA: *Candida albicans* ATCC 60193; SC: *Saccharomyces cerevisiae* RSKK 251; AA: *Ascosphaera apis* 24. NA: not available. PC: positive control. Ampicillin and Fluconazole were used as positive controls for bacteria and fungi, respectively.

**Table 3:** DS: IFD docking score (kcal/mol), ΔG: MM-GBSA binding free energy (kcal/mol) of the **4f**, **4g**, and the cognate ligands.

Compound	DS/ΔG	7NAE	2W95	1M78
**4f**	DS	−12.091	−9.095	−8.867
	ΔG	−84.10	−74.52	−66.49
**4g**	DS	−10.345	−9.070	−8.955
	ΔG	−79.89	−76.20	−74.11
TOP	DS	−10.009	−10.082	-
	ΔG	−88.20	−72.81	-
CLZ	DS	-	-	−8.542
	ΔG	-	-	−54.27

**Table 4:** Non-covalent interaction of the **4f** and **4g** compounds.

PDB ID	Compound	Hydrogen Bonding	Pi-pi Stacking	Hydrophobic
7NAE	**4f**	ALA7, TRP22, ILE94, TRY100	PHE31	ILE5, ILE6, ASN 23, ASP27, LEU28, TRP30, GLY95, GLY96, TRY111, THR113,
	**4g**	ASN 18, SER49, ARG52, ILE94	PHE31	ALA7, ASP27, LEU28, LYS32, THR35, THR46, SER49, ILE50, LEU54, ARG57
	TOP	ILE 4, ILE5, ASP27		ALA6, ALA7, LEU28, TRP30, PHE 31, TRY111, THR113, THR46, SER49, ILE50, ARG 52, LEU54
2W95	**4f**	GLN19		LEU20, TRP22, HIS23, LEU24, ASP27, LEU28, HIE30, ILE31, THR46, SER 49, ILE50
	**4g**	GLN19, TRP22, ASP27		LEU20, HIS23, LEU24, LEU28, ILE31, THR46, SER49, ILE50, PHE92, TRY98
	TOP	ILE5, ASP27, PHE92		VAL6, ALA7, GLN19, LEU20, LEU28, HIE30, ILE31, THR46, SER49, ILE50, LEU54
1M78	**4f**	LYS24, ARG28, GLU32	PHE36	ILE9, VAL10, ALA11, MET25, LYS37, ILE33, MET54, THR58, TRP59, ILE62, LEU69, ARG72, ILE112, TRY118
	**4g**	TRP27, ARG28, GLU32, ILE112		ILE9, VAL10, ALA11, MET25, LEU29, ILE33, PHE36, LYS37, THR58, SER61, ILE62, GLY114, TRY118
	CLZ	ILE9, GLU32, ILE112	PHE36	VAL10, ALA11, MET25, ILE33, TRY35, ILE112, TRY118, LEU131, THR133

**Table 5:** Pharmacokinetic parameters calculated by Qikpro module for **4f** and **4g** compounds.

Parameters	4f	4g
LogHERG	−7.775	−6.591
PCaco	2598.637	2472.974
LogBB	−0.669	−0.649
PMDCK	1388.804	1316.357
PLogKp	0.597	0.016
PLogKhsa	0.716	0.204
CNS	−1	−1
% ABS	100	100
Ro5	1	0
Ro3	1	0

## Data Availability

Data is contained within the article or the Supplementary Materials.

## Supplementary Materials

The following supporting information can be downloaded at: https://www.mdpi.com/article/10.3390/ijms27020742/s1.

## Abbreviations

The following abbreviations are used in this manuscript:

ADME	Absorption, distribution, metabolism, and excretion
AIDS	Acquired immunodeficiency syndrome
AMR	Antimicrobial resistance
ATCC	American Type Culture Collection
CLSI	Clinical and Laboratory Standards Institute
CNS	Central Nervous System
DHFR	Dihydrofolate reductase
FT-IR	Fourier Transform Infrared Spectroscopy
IFD	Induced-fit docking
LCMS-MS	Liquid chromatography–mass spectrometry
MDCK	Madin–Darby canine kidney
MHA	Mueller–Hinton Agar
MIC	Minimum inhibitory concentration
MM-GBSA	Molecular mechanics-generalized born surface area
MM-GBSA	Molecular mechanics-generalized born surface area
MY20	20% dextrose agar
NMR	Nuclear magnetic resonance
PDB	Potato Dextrose Broth
PMDCK	Permeability Madin–Darby Canine Kidney
RCSB	Research Collaboratory for Structural Bioinformatics
RMSD	Root mean square deviation
RSKK	Refik Saydam National Type Culture Collection
WHO	World Health Organization
YPS	Yeast peptone dextrose agar
YPS	Yeast peptone dextrose broth

## References

[1] Ahmed S.K., Hussein S., Qurbani K., Ibrahim R.H., Fareeq A., Mahmood K.A., Mohamed M.G. (2024). Antimicrobial resistance: Impacts, challenges, and future prospects. J. Med. Surg. Public Health.

[2] Irfan M., Almotiri A., AlZeyadi Z.A. (2022). Antimicrobial resistance and its drivers—A review. Antibiotics.

[3] Adefisoye M.A., Olaniran A.O. (2023). Antimicrobial resistance expansion in pathogens: A review of current mitigation strategies and advances towards innovative therapy. JAC-Antimicrob. Resist..

[4] Baquero F. (1997). Gram-positive resistance: Challenge for the development of new antibiotics. J. Antimicrob. Chemother..

[5] Alekshun M.N., Levy S.B. (2007). Molecular mechanisms of antibacterial multidrug resistanceş. Cell.

[6] Rice L.B. (2006). Unmet medical needs in antibacterial therapy. Biochem. Pharmacol..

[7] Sharma P., Ganguly M. (2023). Copper-enhanced fluorescence: A novel platform for the sensing of hydrogen peroxide. New J. Chem..

[8] dos Santos J.E., Dockal E.R., Cavalheiro E.T. (2005). Synthesis and characterization of Schiff bases from chitosan and salicylaldehyde derivatives. Carbohydr. Polym..

[9] Sharma S., Vashishtha M. (2023). Evaluation of optimized molecular structure-antimicrobial and antioxidant efficacy relationship of Schiff bases. Environ. Sci. Pollut. Res..

[10] Iftime M.M., Morariu S., Marin L. (2017). Salicyl-imine-chitosan hydrogels: Supramolecular architecturing as a cross linking method toward multifunctional hydrogels. Carbohydr. Polym..

[11] Ceramella J., Iacopetta D., Catalano A., Cirillo F., Lappano R., Sinicropi M.S. (2022). A review on the antimicrobial activity of Schiff bases: Data collection and recent studies. Antibiotics.

[12] Sethu R.M., Amirthaganesan G. (2004). Structural and spectroscopic studies of substituted Schiff bases. Indian J. Phys..

[13] Schiff H. (1869). Untersuchungen über salicinderivate. Justus Liebigs Ann. Der Chem..

[14] Demir S. (2013). Synthesis, Characterization, and Quantum Chemical Modeling of Transition Metal Complexes of Imidazole-4-Carboxaldehyde–Derived Schiff Bases. Ph.D. Thesis.

[15] Matar S.A., Talib W.H., Mustafa M.S., Mubarak M.S., AlDamen M.A. (2015). Synthesis, characterization, and antimicrobial activity of Schiff bases derived from benzaldehydes and 3,3′-diaminodipropylamine. Arab. J. Chem..

[16] Shen Z.-H., Shi Y.-X., Yang M.-Y., Sun Z.-H., Weng J.-Q., Tan C.-X., Liu X.-H., Li B.-J, Zhao W.-G. (2016). Synthesis, crystal structure, DFT studies and biological activity of a novel Schiff base containing Triazolo[4,3-a]pyridine moiety. Chin. J. Struct. Chem..

[17] Tahtaci H., Er M., Karakurt T., Onaran A. (2017). Synthesis, Structural characterization, and biological evaluation of novel substituted 1,3-thiazole derivatives containing Schiff bases. J. Heterocycl. Chem..

[18] Prasad S., Radhakrishna V., Ravi T.K. (2019). Synthesis, spectroscopic and antibacterial studies of some Schiff bases of 4-(4-bromophenyl)-6-(4-chlorophenyl)-2-aminopyrimidine. Arab. J. Chem..

[19] Zayed E.M., Zayed M.A. (2015). Synthesis of novel Schiff’s bases of highly potential biological activities and their structure investigation. Spectrochim. Acta Part A Mol. Biomol. Spectrosc..

[20] Amnerkar N.D., Bhongade B.A., Bhusari K.P. (2015). Synthesis and biological evaluation of some4-(6-substituted-1,3-benzothiazol-2-yl)amino-1,3-thiazole-2-amines and their Schiff bases. Arab. J. Chem..

[21] Soliman D.H., Eldehna W.M., Ghabbour H.A., Kabil M.M., Abdel-Aziz M.M., Abdel-Aziz H.A.K. (2017). Novel 6-phenylnicotinohydrazide derivatives: Design, synthesis and biological evaluation as a novel class of antitubercular and antimicrobial agents. Biol. Pharm. Bull..

[22] Bringmann G., Dreyer M., Faber J.H., Dalsgaard P.W., Staerk D., Jaroszewski J.W. (2004). Ancistrotanzanine C and related 5,10- and 7,30-coupled naphthylisoquinoline alkaloids from *Ancistrocladus tanzaniensis*. J. Nat. Prod..

[23] Souza A.O.D., Galetti F.C.S., Silva C.L., Bicalho B., Parma M.M., Fonseca S.F. (2007). Antimycobacterial and cytotoxicity activity of synthetic and natural compounds. Quim. Nova.

[24] Guo Z., Xing R., Liu S., Zhong Z., Ji X., Wang L. (2007). Antifungal properties of Schiff bases of chitosan, N-substituted chitosan and quaternized chitosan. Carbohydr. Res..

[25] Boulechfar C., Ferkous H., Delimi A., Djedouani A., Kahlouche A., Boublia A., Benguerba Y. (2023). Schiff bases and their metal Complexes: A review on the history, synthesis, and applications. Inorg. Chem. Commun..

[26] Aslam M., Anis I., Mehmood R., Iqbal L., Iqbal S., Khan I., Chishti M.S., Perveen S. (2016). Synthesis and biological activities of 2-aminophenol-based Schiff bases and their structure–activity relationship. Med. Chem. Res..

[27] Khan M., Khan A., Taha M., Salar U., Hameed A., Ismail N.H., Jamil W., Saad S.M., Perveen S., Kashif S.M. (2015). Synthesis of 4-amino-1,5-dimethyl-2-phenylpyrazolone derivatives and their antioxidant activity. J. Chem. Soc. Pak..

[28] Prathima B., Rao Y.S., Reddy S.A., Reddy Y.P., Reddy A.V. (2010). Copper(II) and nickel(II) complexes of benzyloxybenzaldehyde-4-phenyl-3-thiosemicarbazone: Synthesis, characterization and biological activity. Spectrochim. Acta.

[29] Clinical Laboratory Standards Institute (2006). Performance Standards for Antimicrobial Disk Susceptibility Tests.

[30] Irvine J.D., Takahashi L., Lockhart K., Cheong J., Tolan J.W., Selick H.E., Grove J.R. (1999). MDCK (Madin–Darby canine kidney) cells: A tool for membrane permeability screening. J. Pharm. Sci..

[31] Jorge J., Del Pino Santos K.F., Timoteo F., Piva Vasconcelos R.R., Ignacio Ayala Caceres O., Juliane Arantes Granja I., Rafique J. (2024). Recent advances on the antimicrobial activities of Schiff bases and their metal complexes: An updated overview. Curr. Med. Chem..

[32] Aftab H., Ullah S., Khan A., Al-Rashida M., Islam T., Dahlous K.A., Shafiq Z. (2024). Design, synthesis, in vitro and in silico studies of novel piperidine derived thiosemicarbazones as inhibitors of dihydrofolate reductase. Sci. Rep..

[33] Naylor M.R., Ly A.M., Handford M.J., Ramos D.P., Pye C.R., Furukawa A., Lokey R.S. (2018). Lipophilic permeability efficiency reconciles the opposing roles of lipophilicity in membrane permeability and aqueous solubility. J. Med. Chem..

[34] Reynaldi F.J., Lucia M., Garcia M.L.G. (2015). *Ascosphaera apis*, the entomopathogenic fungus affecting larvae of native bees (*Xylocopa augusti*): First report in South America. Rev. Iberoam. Micol..

[35] Rufnengo S., Pena N.I., Clemente G., Palacio M.A., Escande A. (2020). Suitability of culture media for the production of ascospoRes. and maintenance of *Ascosphaera apis*. J. Apic. Res..

[36] Jensen A.B., Aronstein K., Flores J.M., Vojvodic S., Palacio M.A., Spivak M. (2013). Standard methods for fungal brood disease research. J. Apic. Res..

[37] Rosdee S., Wisessombat S., Tayeh M., Malakul R., Phanaksri T., Sianglum W. (2025). Antibacterial activity of the endophytic fungal extracts and synergistic effects of combinations of ethylenediaminetetraacetic acid (EDTA) against *Pseudomonas aeruginosa* and *Escherichia coli*. PeerJ.

[38] Mraz P., Zabka M., Hostickova I., Kopecky M., Bohata A., Tomcala A., Hybl M. (2023). Effect of selected botanical compounds on *Ascosphaera apis* and *Apis mellifera*. Ind. Crops Prod..

[39] Schrödinger (2020). Schrödinger Softwere.

[40] Nabuurs S.B., Wagener M., De Vlieg J. (2007). A flexible approach to induced fit docking. J. Med. Chem..

[41] Genheden S., Ryde U. (2015). The MM/PBSA and MM/GBSA methods to estimate ligand-binding affinities. Expert Opin. Drug Discov..

