# Supplementary material for: Synthesis, Antimicrobial Evaluation, and Molecular Docking Analysis of Novel Schiff Bases Derived from Isatoic Anhydride and Salicylaldehyde
Source: Int J Mol Sci. 2026 Jan 11;27(2):742. doi: 10.3390/ijms27020742 (PMC12840646; doi:10.3390/ijms27020742)
Supplement: Supplementary file 1 [file ijms-27-00742-s001.zip › ijms-4080252-supplementary.pdf]

Synthesis, Antimicrobial Evaluation, and Molecular Docking Analysis of Novel Schiff Bases Derived from Isatoic Anhydride and Salicylaldehyde

Turgay Tunç [1,*] and Yaşar Köse [1]

[1] Department of Chemical Engineering, Faculty of Engineering and Architecture, Kırşehir Ahi Evran University, 40100, Kırşehir, Türkiye; ttunc@ahievran.edu.tr (T .T.); yasar-_kose40@hotmail.com

* Correspondence: ttunc@ahievran.edu.tr; Tel.: +90 386 280 22 00

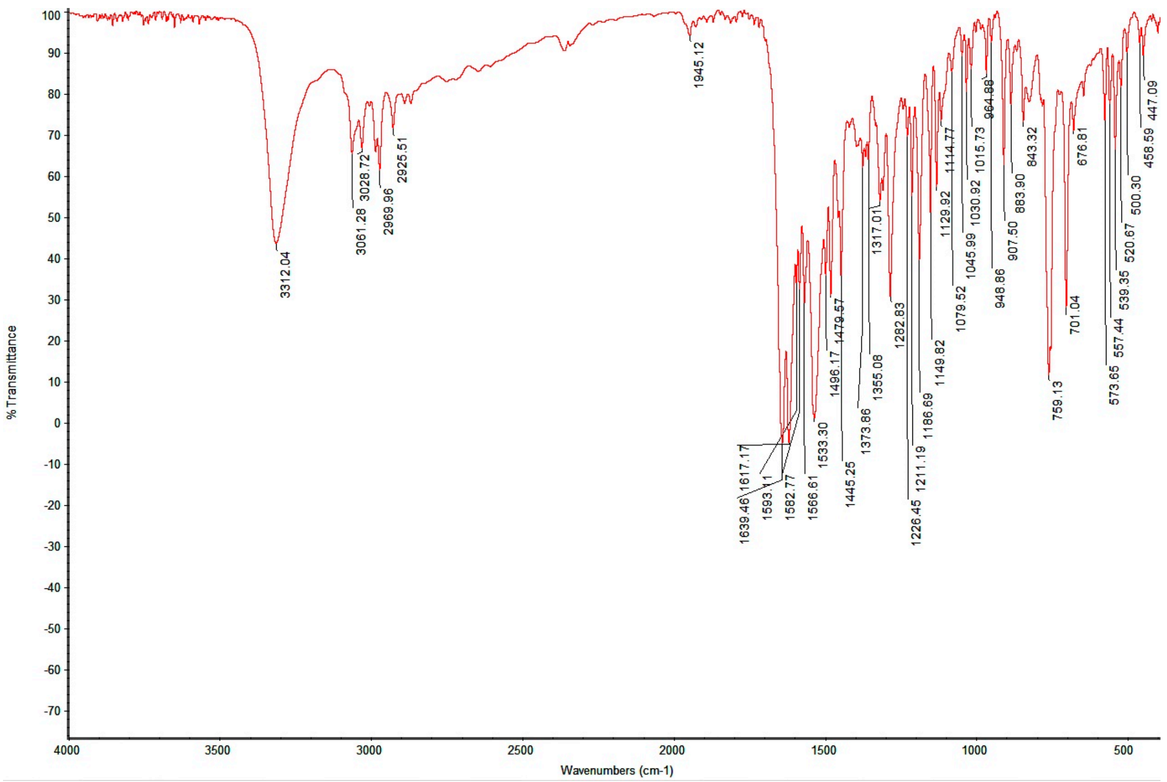

Supplementary figure S1. FTIR spectrum of 4a.

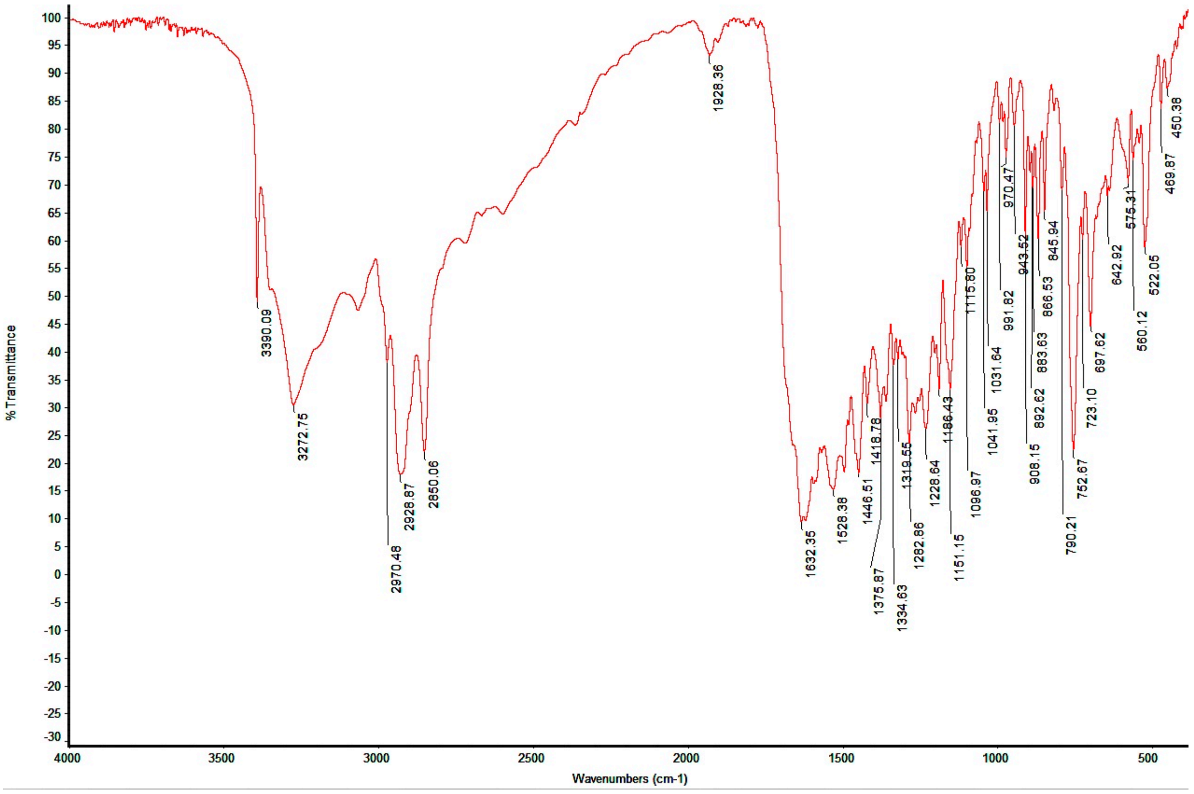

Supplementary figure S2. FTIR spectrum of 4b.

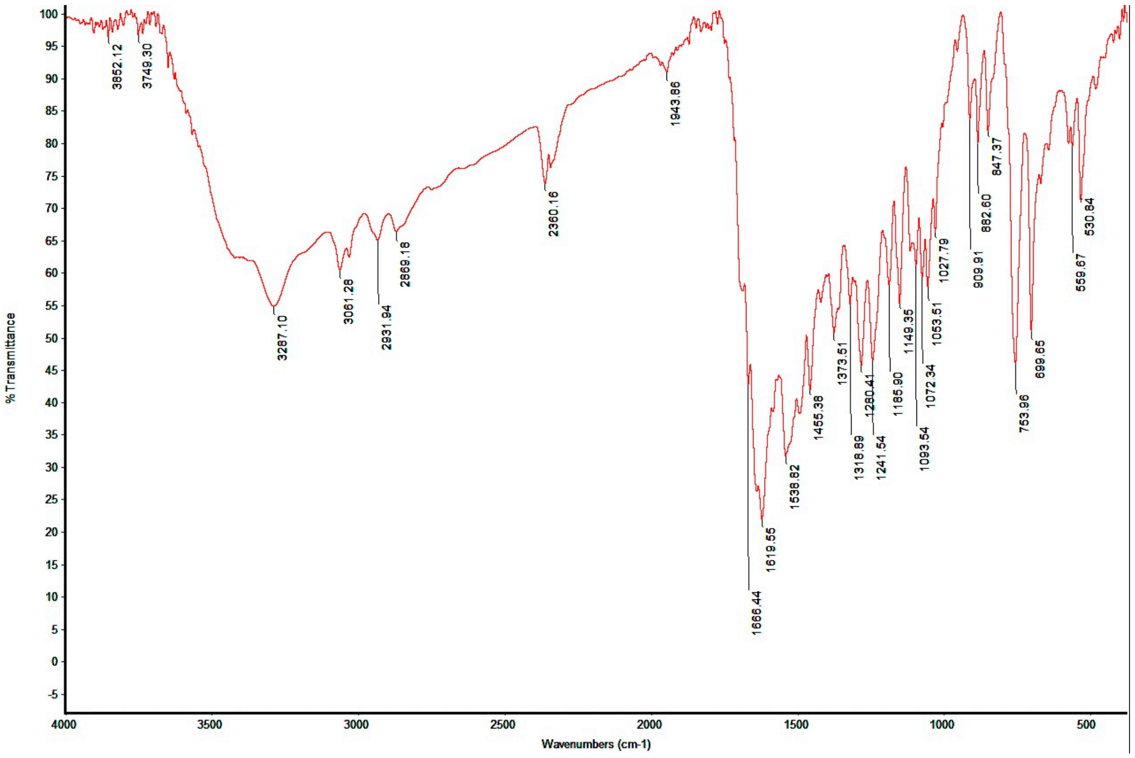

Supplementary figure S3. FTIR spectrum of 4c.

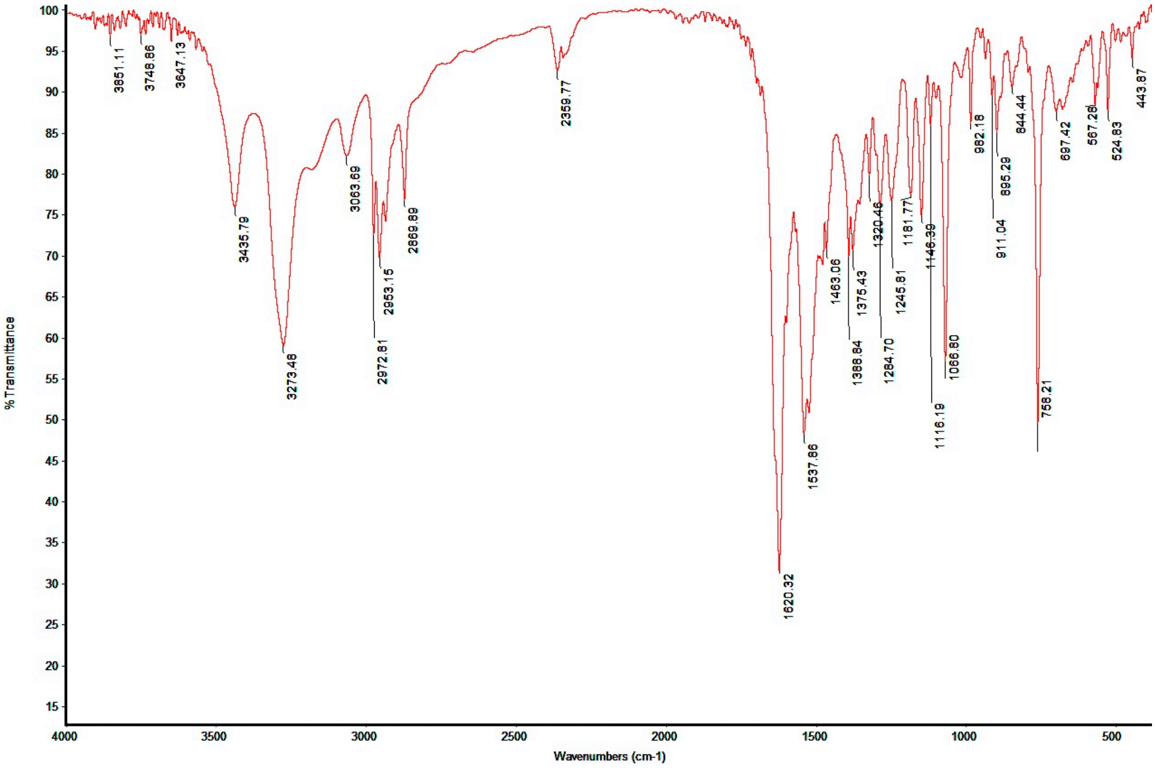

Supplementary figure S4. FTIR spectrum of 4d.

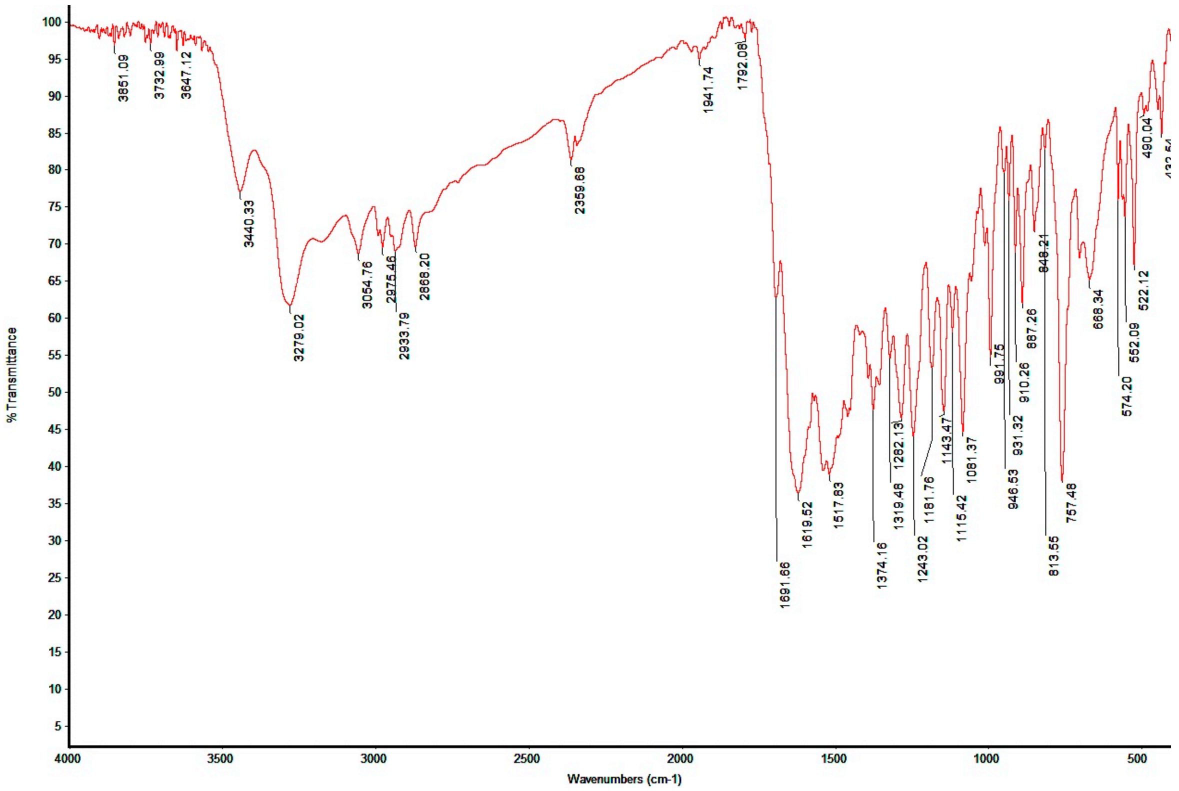

Supplementary figure S5. FTIR spectrum of 4e.

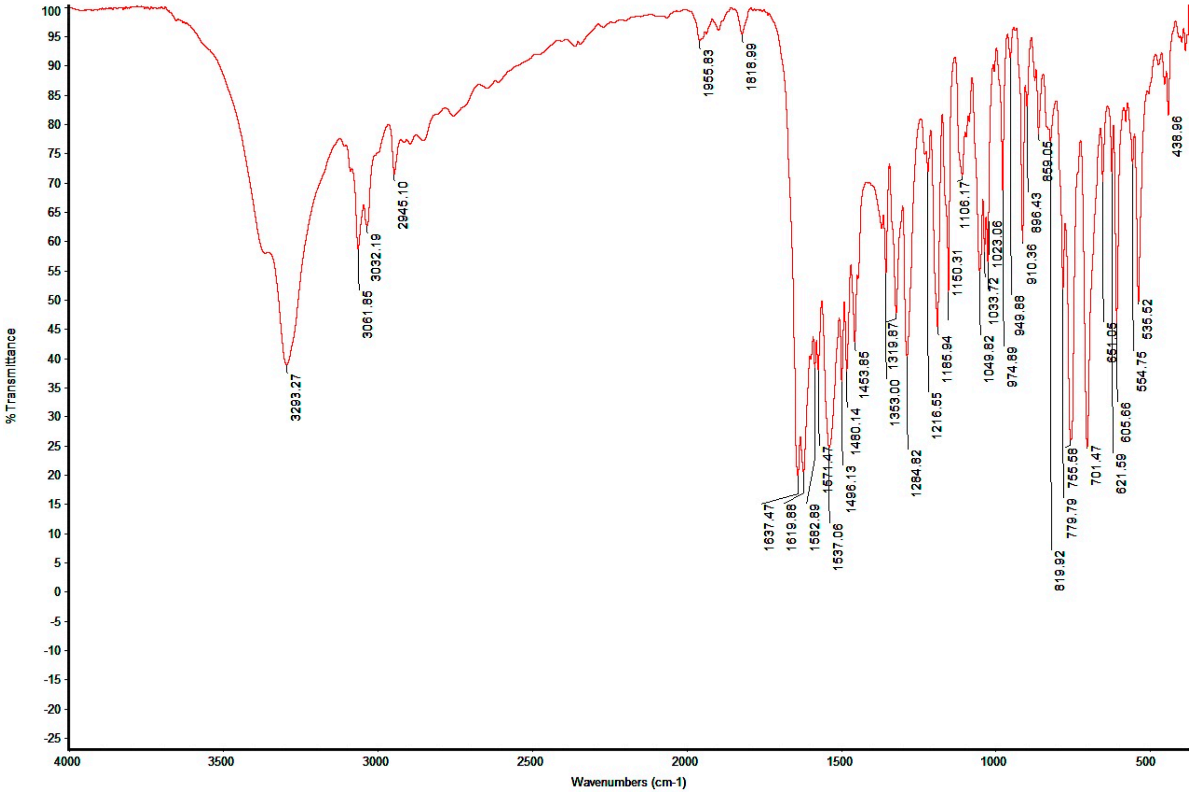

Supplementary figure S6. FTIR spectrum of 4f.

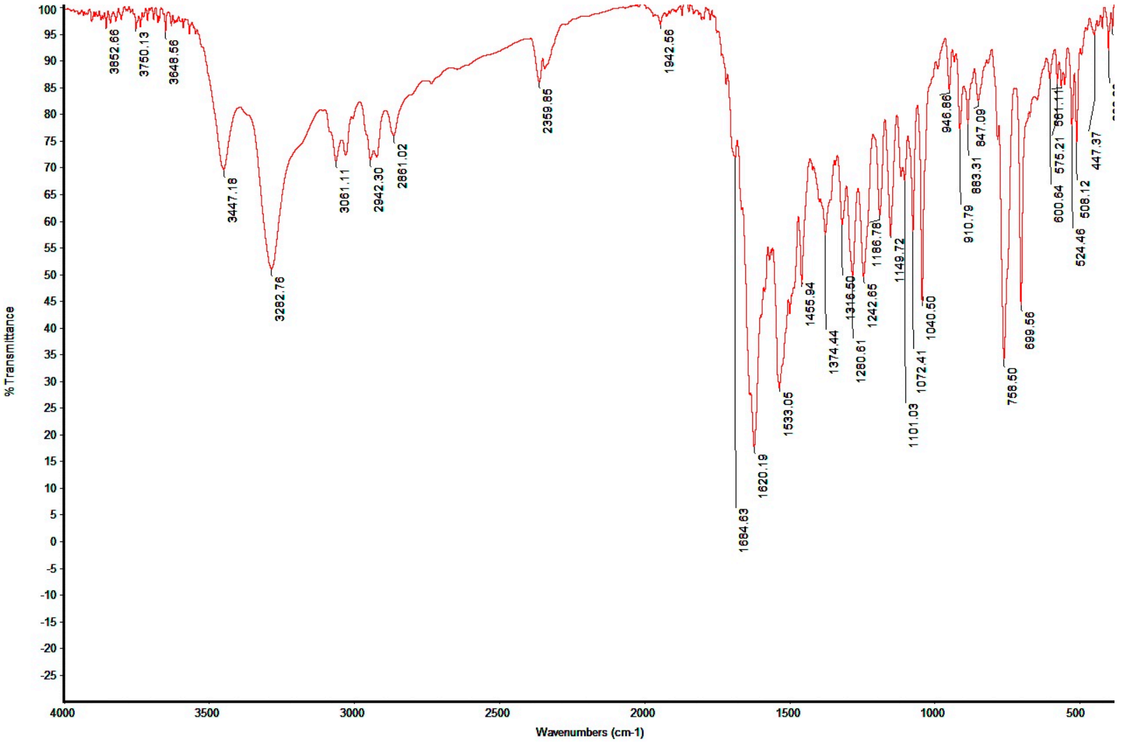

Supplementary figure S7. FTIR spectrum of 4g.

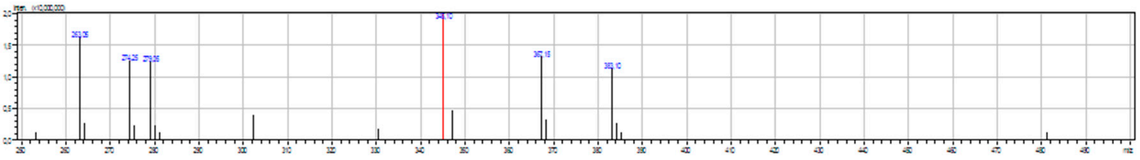

[MS Spectrum]
# of Peaks 18
Raw Spectr [0,101],(scan:[7])
Backgroun No Background Spectrum
Base Peak m/z 345,10 (Inten : 20.000.000)

m/z	Absolute In	Relative Intensity
253,1	1128284	5,64
263,05	16336882	81,68
264,1	2673457	13,37
274,25	12522936	62,61
275,3	2185929	10,93
279,05	12365897	61,83
280,1	2191187	10,96
281,1	1195457	5,98
302,3	3863615	19,32
330,35	1691910	8,46
345,1	20000000	100
347,2	4636217	23,18
367,15	13160795	65,8
368,15	3138699	15,69
383,1	11386849	56,93
384,15	2592443	12,96
385,15	1207441	6,04
481,25	1248856	6,24

Event 1

Supplementary figure S8. LCMS spectrum of 4a.

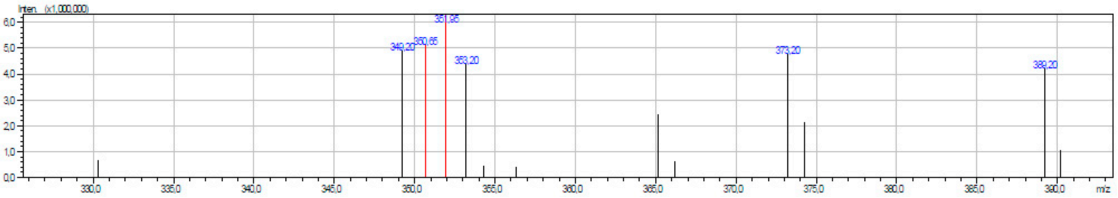

[MS Spectrum]
# of Peaks 24
Raw Spectr [0,034->0,169],(scan:[3->11])
Backgroun [0,000->0,947],(scan:[1->57])
Base Peak m/z 351,95 (Inten : 6.348.561)

m/z	Absolute Ir	Relative Intensity
259,15	749617	11,81
274,25	3374687	53,16
275,35	539495	8,5
289,2	585321	9,22
291,15	478750	7,54
302,3	1213950	19,12
303,2	1328342	20,92
330,3	657861	10,36
349,2	4900474	77,19
350,65	5117841	80,61
351,95	6348561	100
353,2	4373066	68,88
354,3	431503	6,8
356,35	375925	5,92
365,15	2432890	38,32
366,2	610249	9,61
373,2	4773571	75,19
374,25	2116109	33,33
389,2	4210958	66,33
390,2	1044793	16,46
418,3	443230	6,98
478,4	1437565	22,64
479,35	507087	7,99
492,25	668662	10,53

Event 1

Supplementary figure S9. LCMS spectrum of 4b.

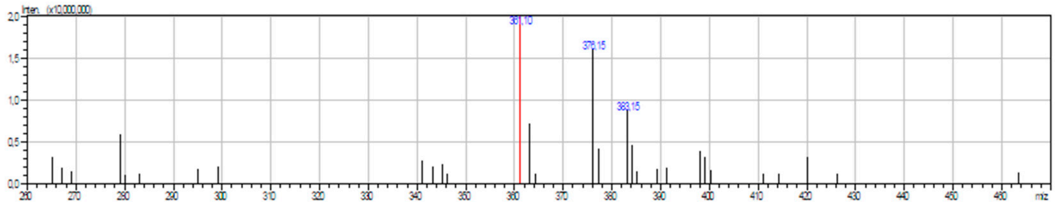

[MS Spectrum]
# of Peaks 30
Raw Spectr [0,101],(scan:[7])
Backgroun No Background Spectrum
Base Peak m/z 361,10 (Inten : 20.000.000)

m/z	Absolute Ir	Relative Intensity
265,1	3170611	15,85
267,1	1827423	9,14
269,1	1409115	7,05
279,1	5855560	29,28
280,1	1088065	5,44
283,1	1117841	5,59
295,1	1777143	8,89
299,15	2019209	10,1
341,15	2696422	13,48
343,2	2015472	10,08
345,15	2330321	11,65
346,15	1118821	5,59
361,1	20000000	100
363,15	7215902	36,08
364,2	1195739	5,98
376,15	16143931	80,72
377,2	4238565	21,19
383,15	8815355	44,08
384,15	4651191	23,26
385,2	1482503	7,41
389,2	1708082	8,54
391,2	1911250	9,56
398,2	3862673	19,31
399,15	3242621	16,21
400,2	1543742	7,72
411,15	1126826	5,63
414,15	1173361	5,87
420,15	3123716	15,62
426,15	1121342	5,61
463,3	1287408	6,44

Event 1

Supplementary figure S10. LCMS spectrum of 4c.

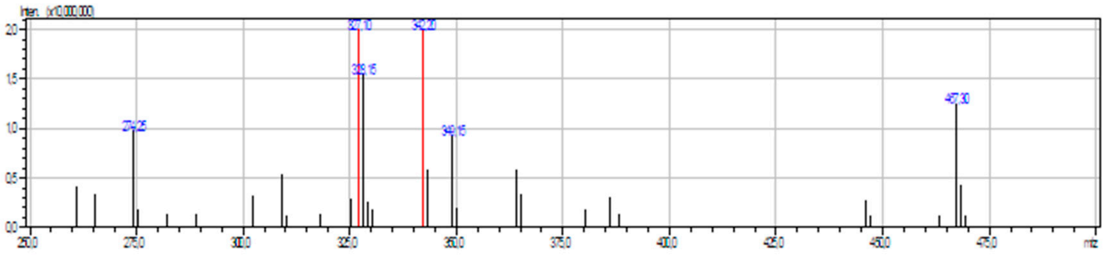

[MS Spectrum]

# of Peaks	30	
Raw Spectr	[0,101],(scan:[7])	
Backgroun	No Background Spectrum	
Base Peak	m/z 327,10 (Inten : 20.000.000)	
m/z	Absolute In	Relative Intensity
261,1	4030932	20,15
265,15	3239175	16,2
274,25	9735378	48,68
275,25	1733646	8,67
282,25	1252109	6,26
289,1	1354842	6,77
302,3	3211988	16,06
309,15	5336630	26,68
310,2	1108063	5,54
318,25	1222224	6,11
325,15	2856232	14,28
327,1	20000000	100
328,15	15450272	77,25
329,2	2460497	12,3
330,3	1687993	8,44
342,2	20000000	100
343,15	5770365	28,85
349,15	9205991	46,03
350,2	1928698	9,64
364,15	5707266	28,54
365,15	3329857	16,65
380,15	1705895	8,53
386,15	2964955	14,82
388,25	1288580	6,44
446,2	2669843	13,35
447,2	1052890	5,26
463,3	1060787	5,3
467,3	12436356	62,18
468,3	4253381	21,27
469,3	1140566	5,7
Event	1	

Supplementary figure S11. LCMS spectrum of 4d.

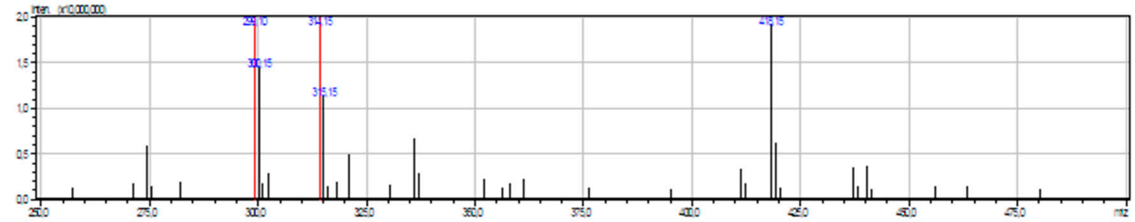

[MS Spectrum]		
# of Peaks	35	
Raw Spectr	[0,068],(scan:[5])	
Backgroun	No Background Spectrum	
Base Peak	m/z 299,10 (Inten : 20.000.000)	
m/z	Absolute Ir	Relative Intensity
257,1	1174740	5,87
271,1	1775868	8,88
274,25	5820826	29,1
275,3	1308320	6,54
282,25	1837952	9,19
299,1	20000000	100
300,15	14501608	72,51
301,15	1632234	8,16
302,3	2742942	13,71
314,15	20000000	100
315,15	11415606	57,08
316,15	1422304	7,11
318,2	1795711	8,98
321,1	4809544	24,05
330,3	1465298	7,33
336,15	6576601	32,88
337,15	2885765	14,43
352,1	2172718	10,86
356,35	1220458	6,1
358,2	1689391	8,45
361,25	2118162	10,59
376,1	1240229	6,2
395,3	1083856	5,42
411,2	3332356	16,66
412,2	1678465	8,39
418,15	19148154	95,74
419,2	6165966	30,83
420,2	1258952	6,29
437,25	3385984	16,93
438,3	1323296	6,62
440,2	3631220	18,16
441,2	1026901	5,13
456,15	1440400	7,2
463,3	1433187	7,17
480,15	1003286	5,02
Event	1	

Supplementary figure S12. LCMS spectrum of 4e.

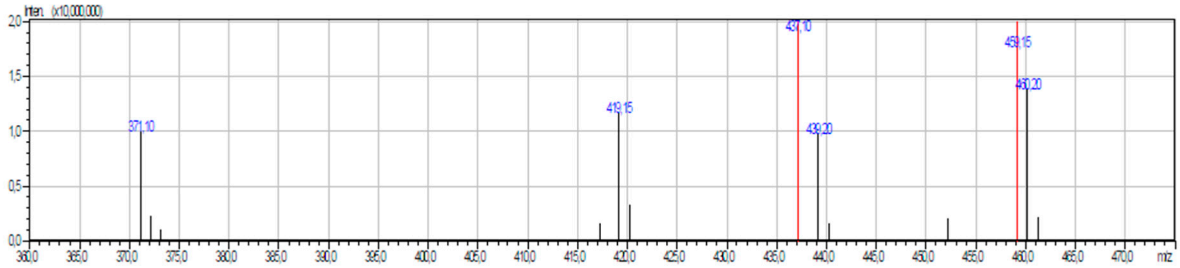

[MS Spectrum]		
# of Peaks	14	
Raw Spectr	[0,101],(scan:[7])	
Backgroun	No Background Spectrum	
Base Peak	m/z 459,15 (Inten : 20.000.000)	
m/z	Absolute Ir	Relative Intensity
371,1	9946242	49,73
372,1	2242970	11,21
373,1	1031810	5,16
417,2	1560728	7,8
419,15	11635149	58,18
420,2	3272944	16,36
437,1	20000000	100
439,2	9793263	48,97
440,2	1621393	8,11
452,15	1965806	9,83
459,15	20000000	100
460,2	13830627	69,15
461,2	2094463	10,47
475,15	8435340	42,18
Event	1	

Supplementary figure S13. LCMS spectrum of 4f.

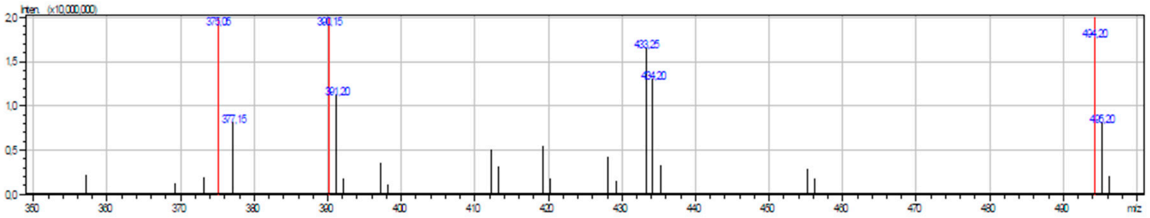

[MS Spectrum]		
# of Peaks	24	
Raw Spectr	[0,068],(scan:[5])	
Backgroun	No Background Spectrum	
Base Peak	m/z 375,05 (Inten : 20.000.000)	
m/z	Absolute Ir	Relative Intensity
357,15	2184025	10,92
369,2	1151965	5,76
373,15	1926869	9,63
375,05	20000000	100
377,15	8126791	40,63
390,15	20000000	100
391,2	11218686	56,09
392,2	1717120	8,59
397,15	3465092	17,33
398,2	1053449	5,27
412,2	5000635	25
413,15	3122009	15,61
419,25	5442772	27,21
420,25	1710280	8,55
428,15	4162479	20,81
429,2	1488110	7,44
433,25	16489129	82,45
434,2	12987350	64,94
435,2	3243711	16,22
455,2	2806201	14,03
456,2	1760288	8,8
494,2	20000000	100
495,2	8107166	40,54
496,25	2012483	10,06
Event	1	

Supplementary figure S14. LCMS spectrum of 4g.

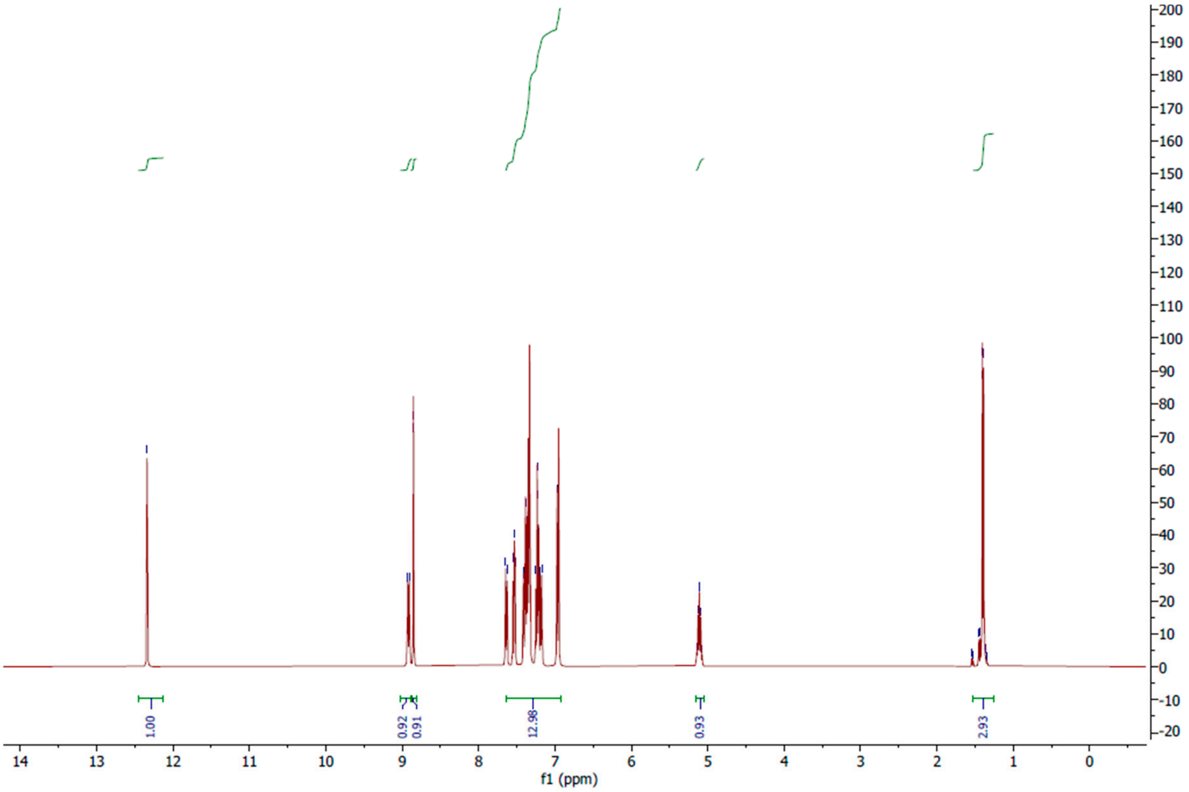

Supplementary figure S15. ^{1}H-NMR spectrum of 4a.

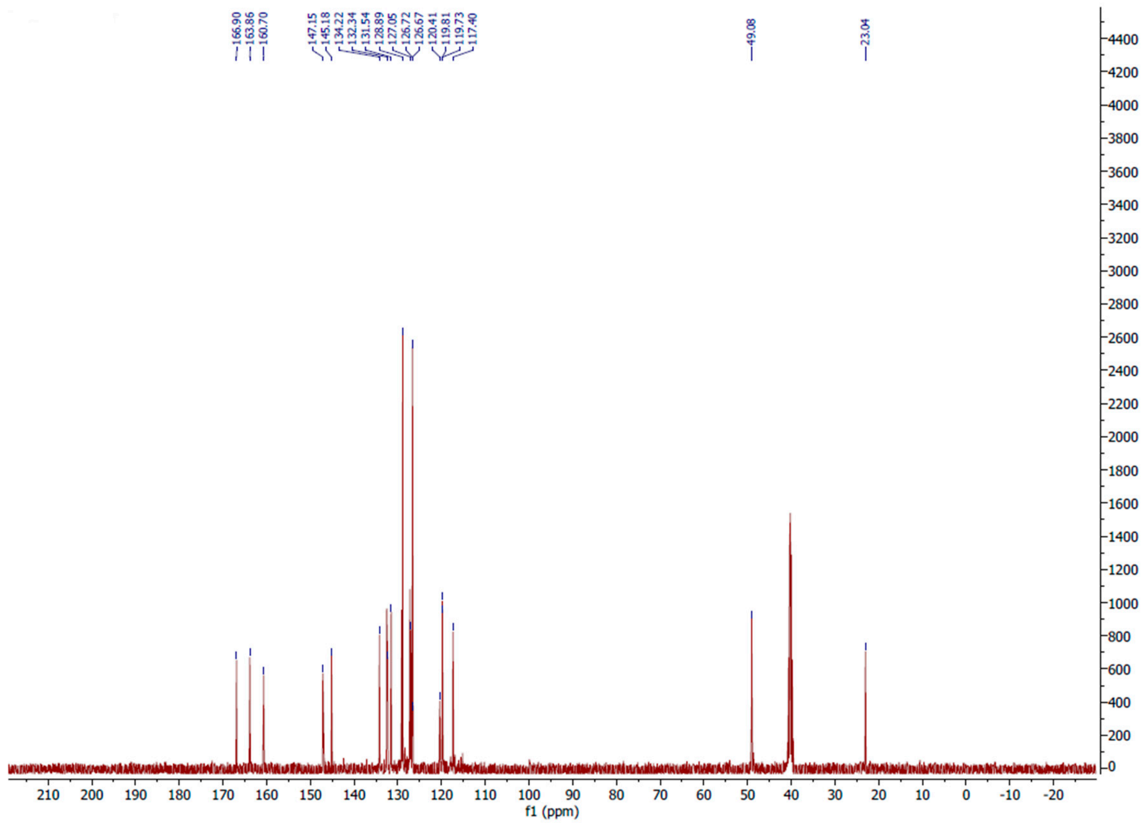

Supplementary figure S16. ^{13}C-NMR spectrum of 4a.

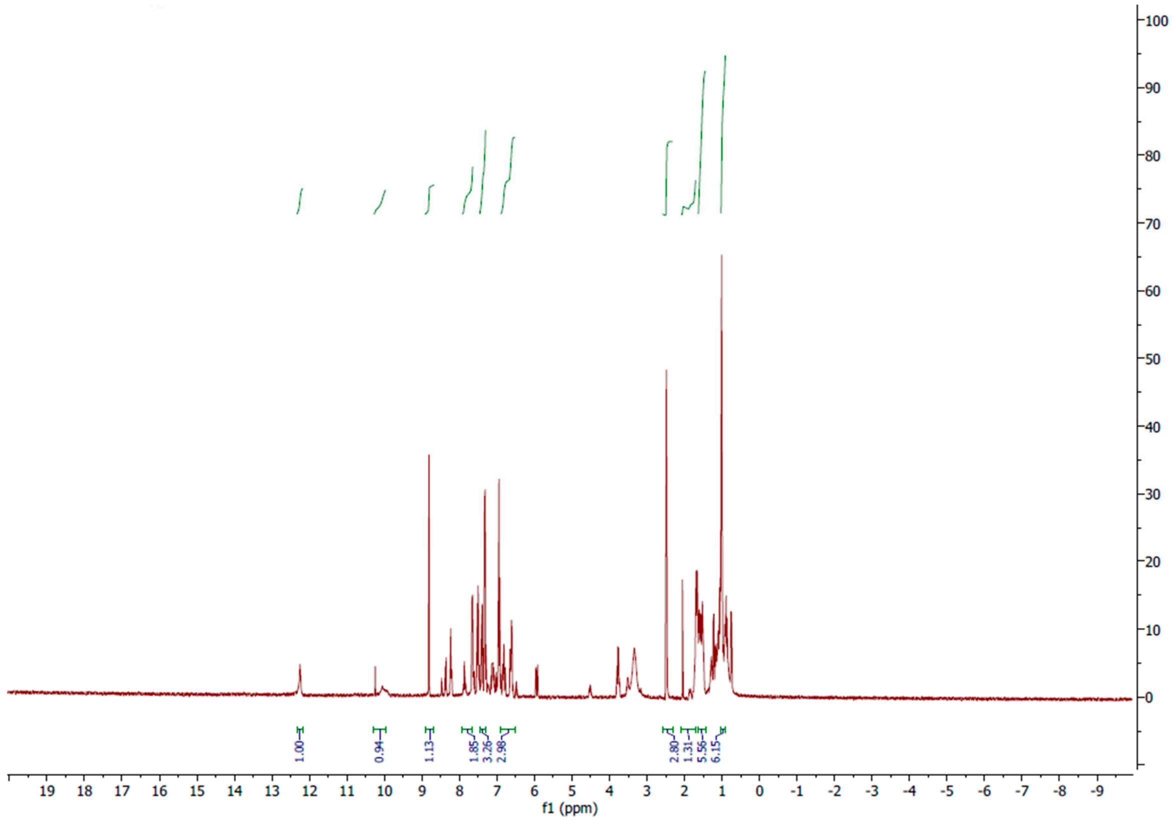

Supplementary figure S17. ^{1}H-NMR spectrum of 4b.

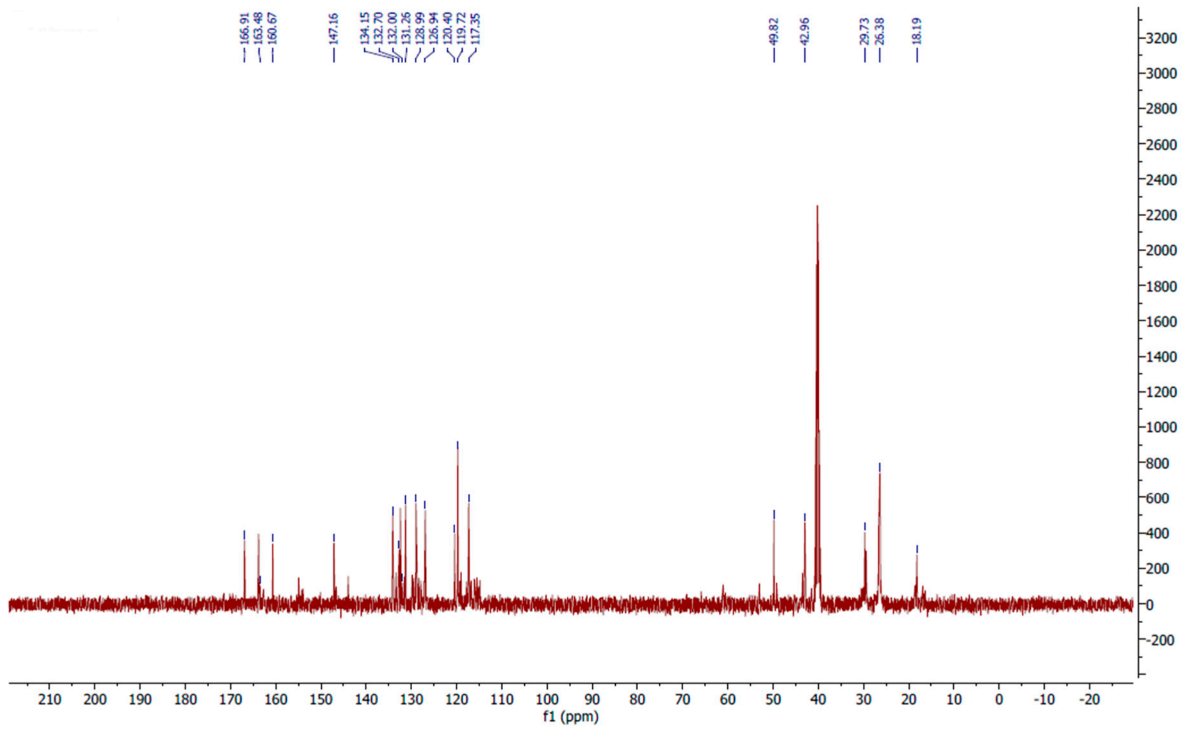

Supplementary figure S18. ^{13}C-NMR spectrum of 4b.

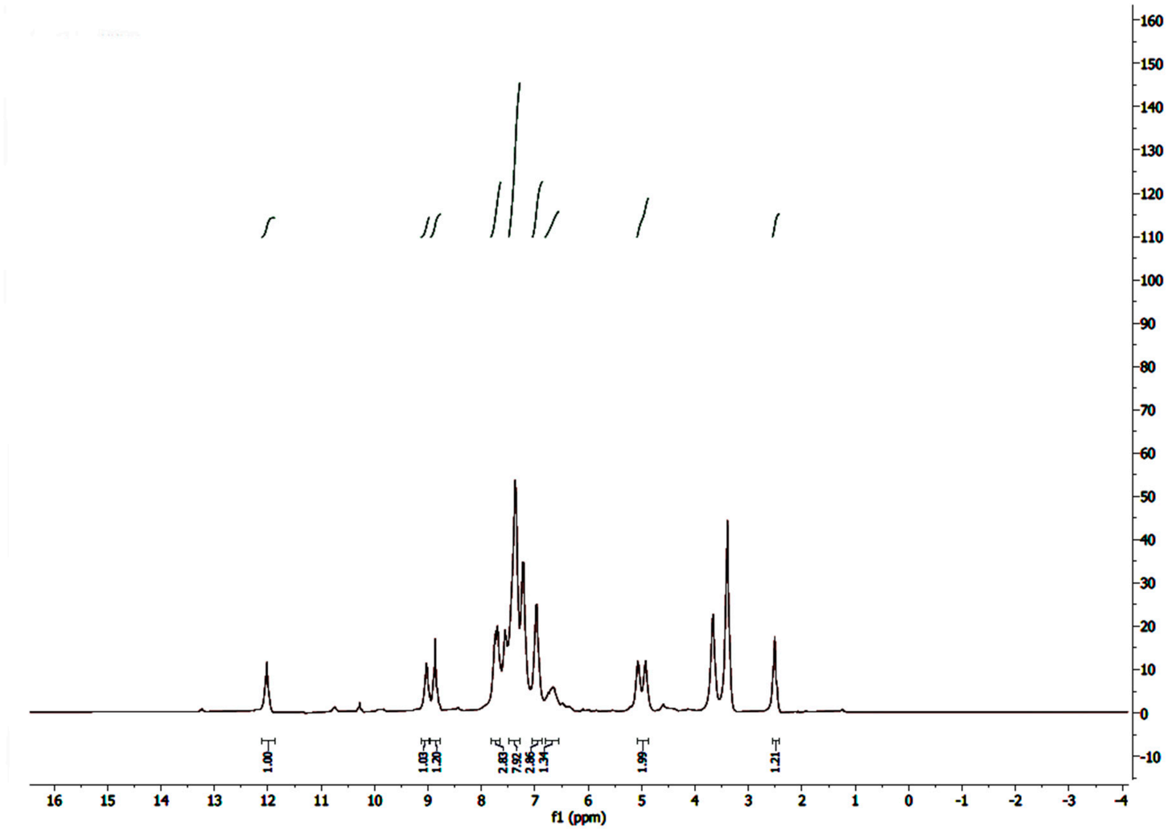

Supplementary figure S19. ^{1}H-NMR spectrum of 4c.

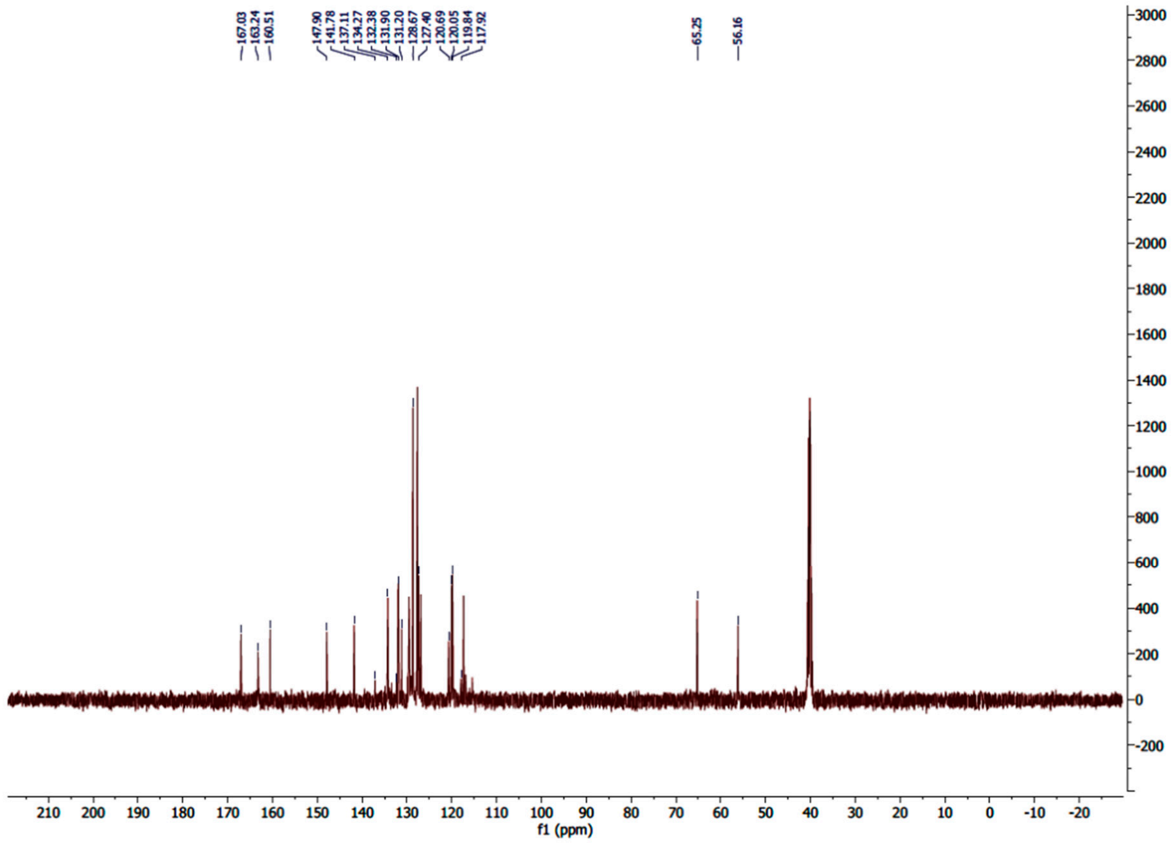

Supplementary figure S20. ^{13}C-NMR spectrum of 4c.

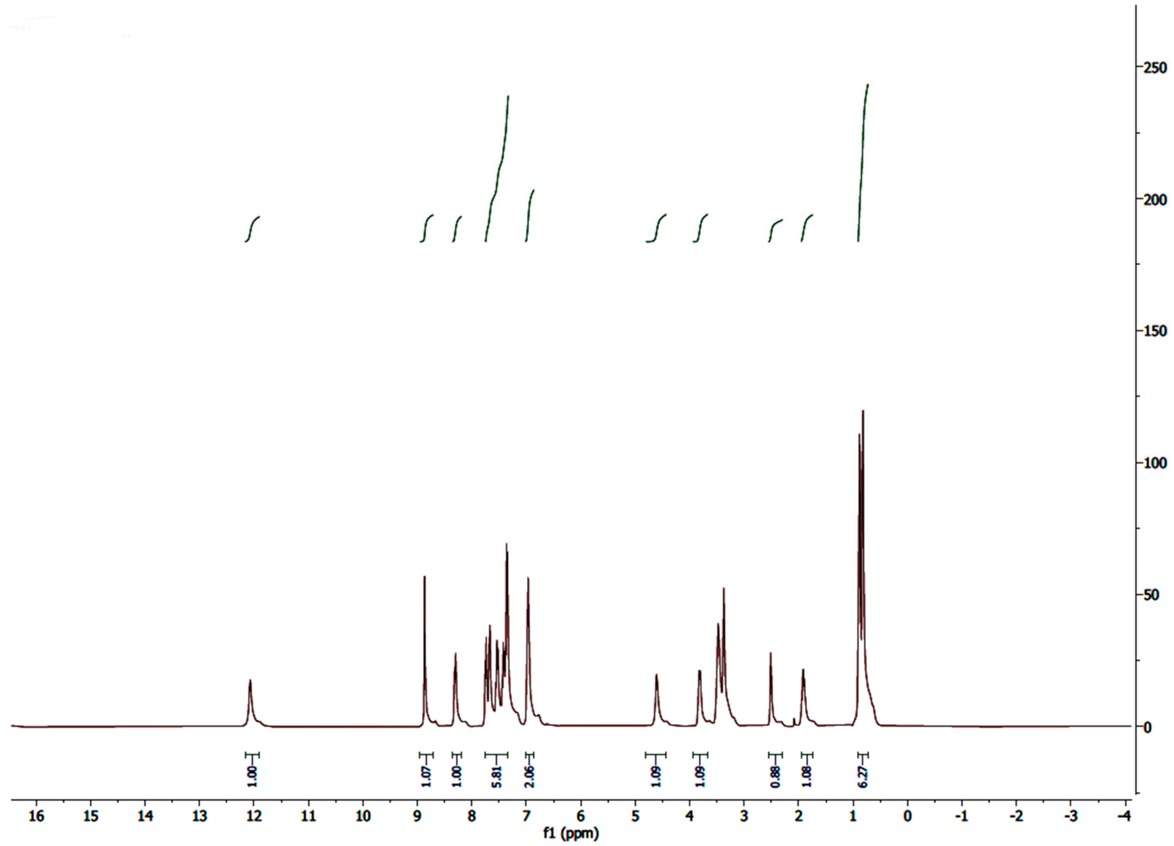

Supplementary figure S21. ^{1}H-NMR spectrum of 4d.

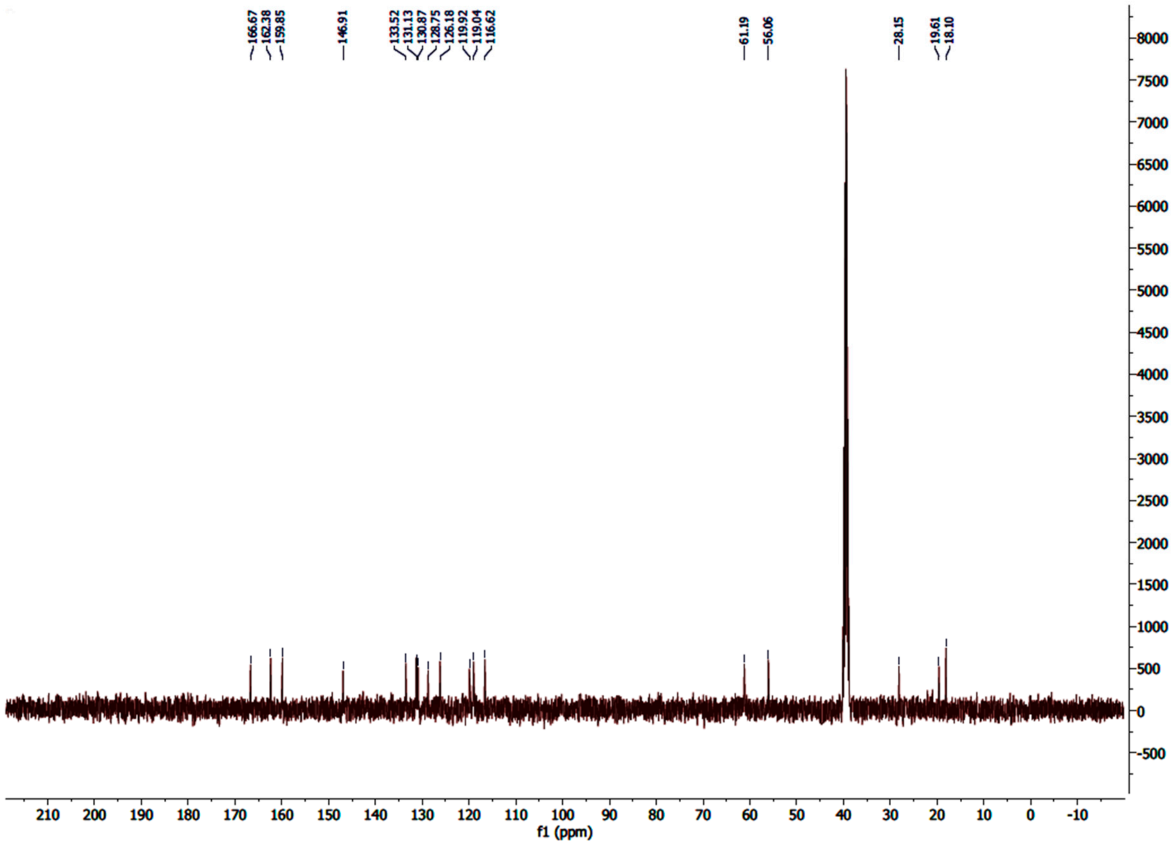

Supplementary figure S22. ^{13}C-NMR spectrum of 4d.

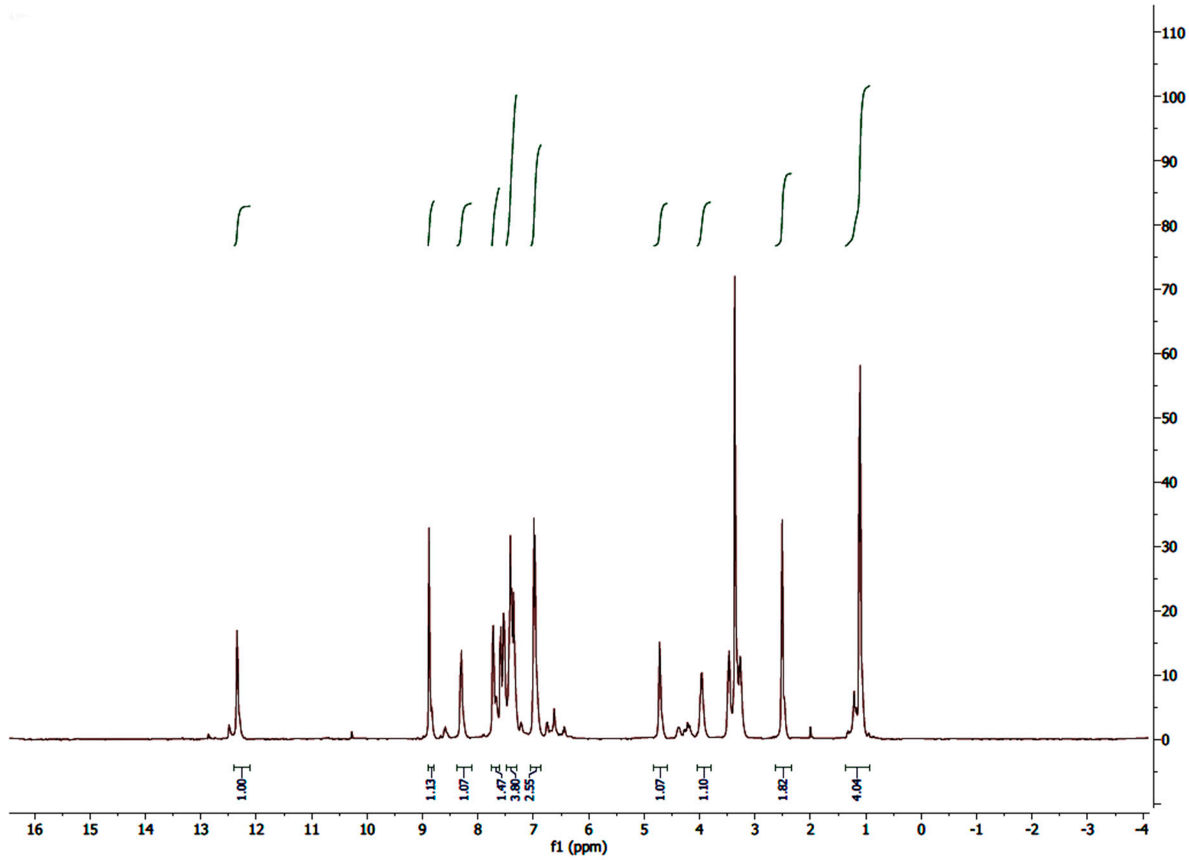

Supplementary figure S23. ^{1}H-NMR spectrum of 4e.

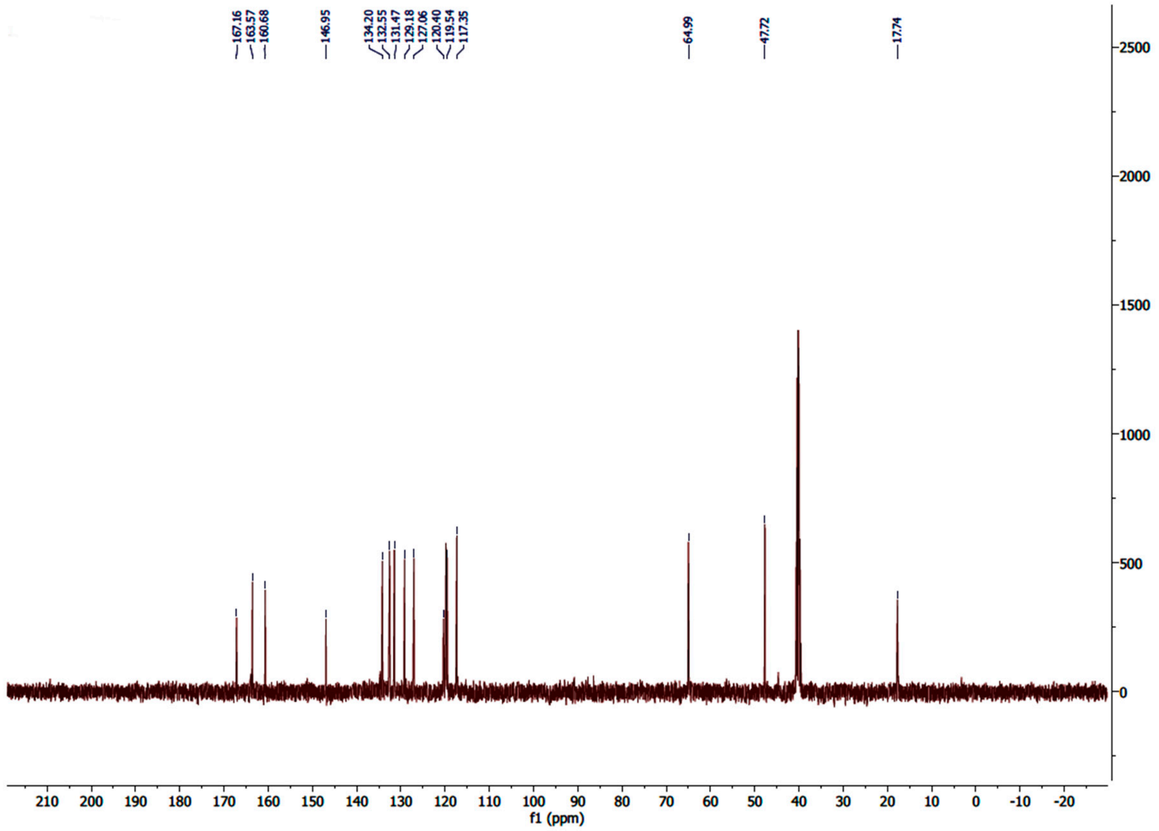

Supplementary figure S24. ^{13}C-NMR spectrum of 4e.

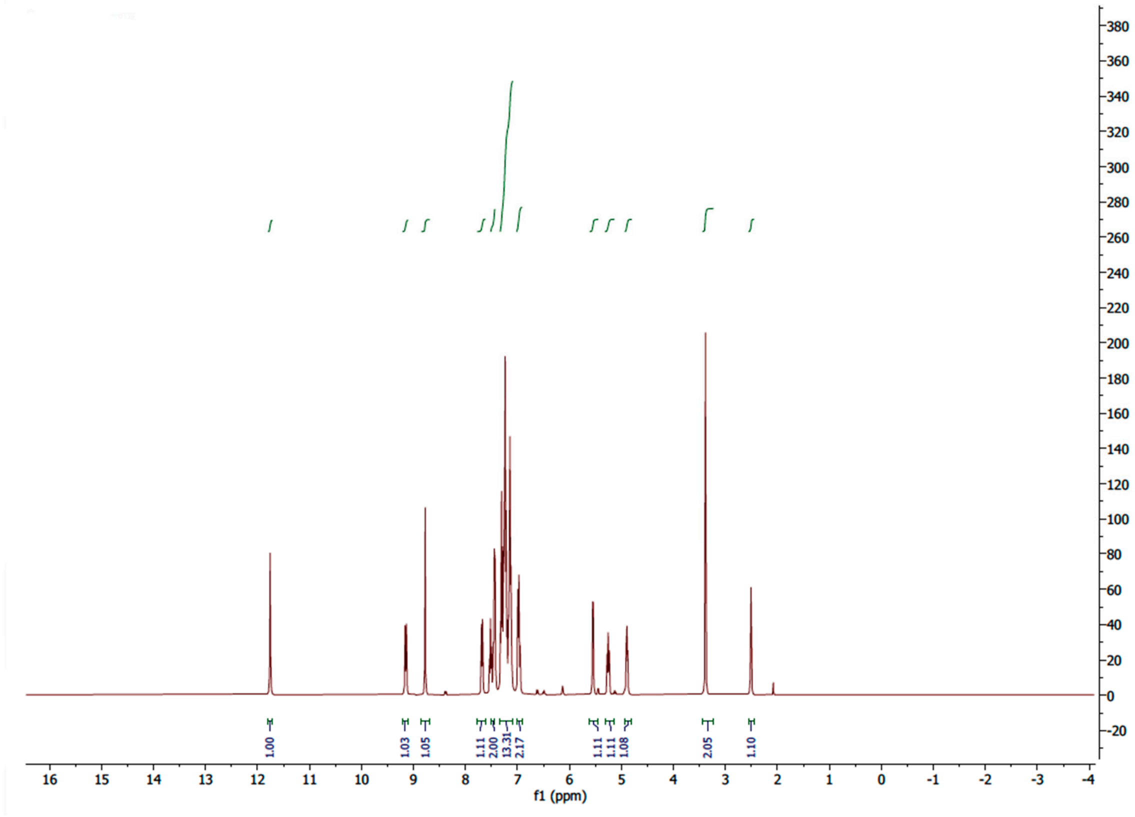

Supplementary figure S25. ^{1}H-NMR spectrum of 4f.

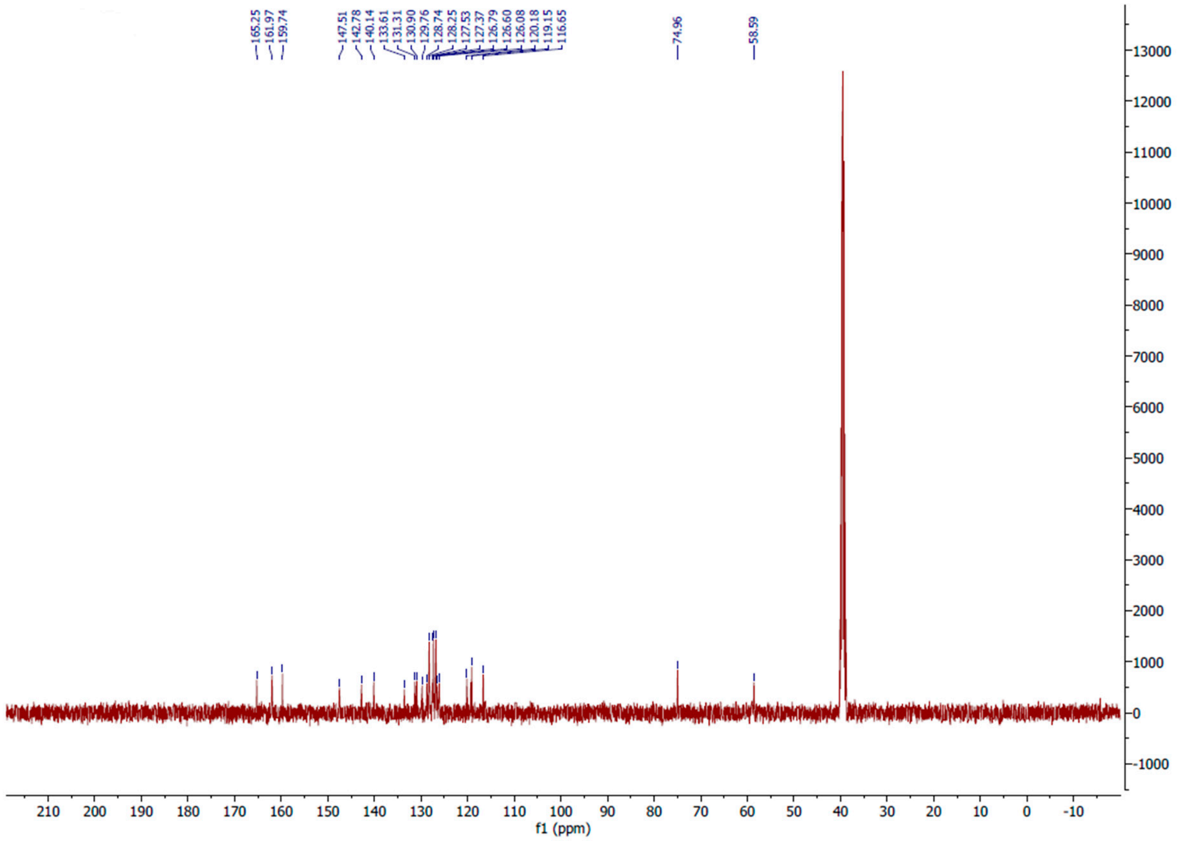

Supplementary figure S26. ^{13}C-NMR spectrum of 4f.

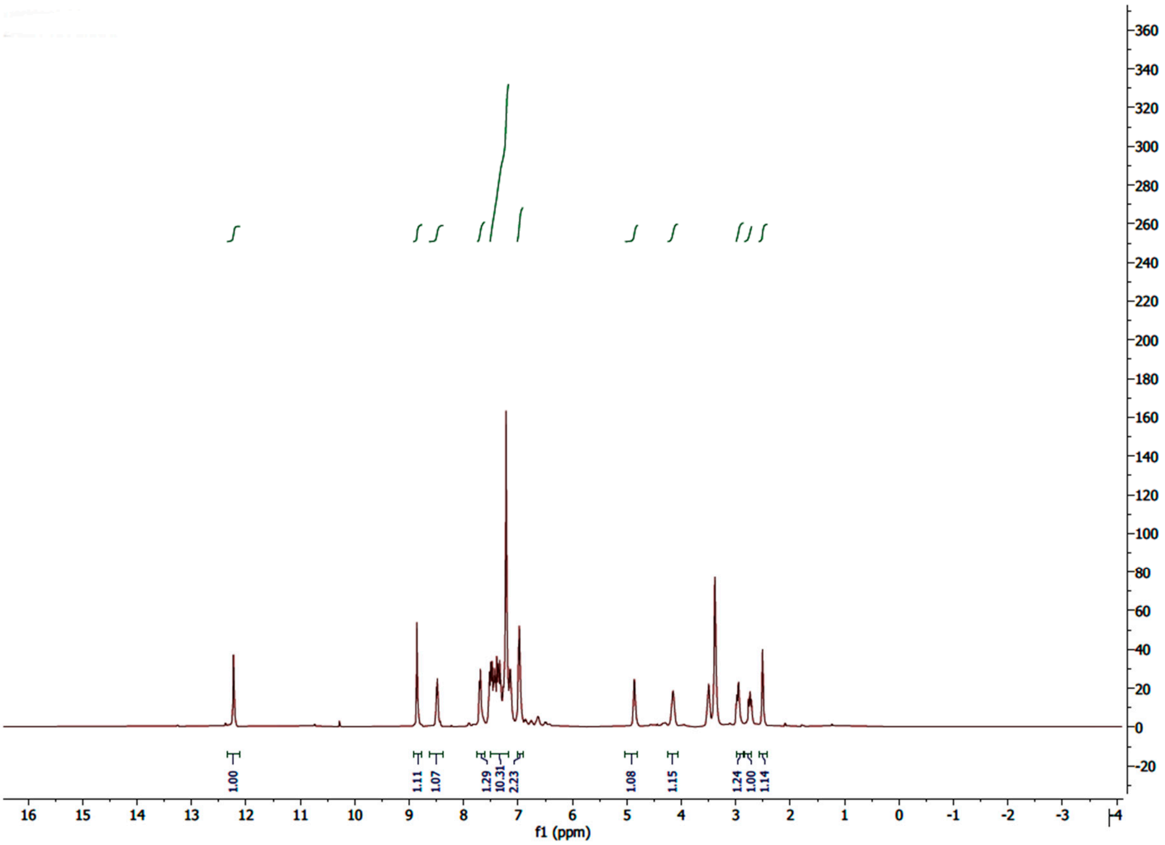

Supplementary figure S27. ^{1}H-NMR spectrum of 4g.

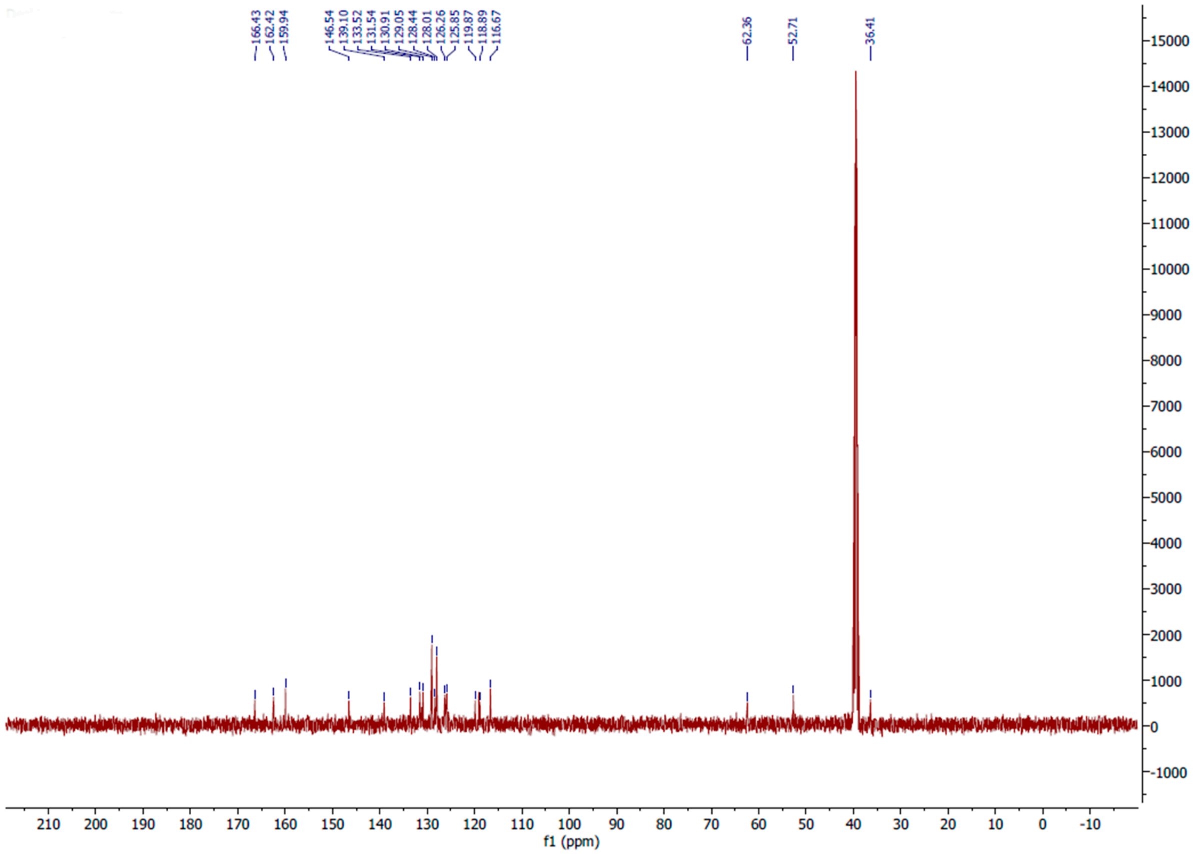

Supplementary figure S28. ^{13}C-NMR spectrum of 4g.
